# Functional Metagenomics Reveals Novel Pathways of Prebiotic Breakdown by Human Gut Bacteria

**DOI:** 10.1371/journal.pone.0072766

**Published:** 2013-09-16

**Authors:** Davide A. Cecchini, Elisabeth Laville, Sandrine Laguerre, Patrick Robe, Marion Leclerc, Joël Doré, Bernard Henrissat, Magali Remaud-Siméon, Pierre Monsan, Gabrielle Potocki-Véronèse

**Affiliations:** 1 Université de Toulouse, Institut National des Sciences Appliquées, Université Paul Sabatier, Institut National Polytechnique, Ingénierie des Systèmes Biologiques et des Procédés, Toulouse, France; 2 UMR5504, UMR792 Ingénierie des Systèmes Biologiques et des Procédés, Centre National de la Recherche Scientifique, Institut National de la Recherche Agronomique, Toulouse, France; 3 LibraGenSA, Toulouse, France; 4 Institut National de la Recherche Agronomique, Micalis, UMR1319, Jouy en Josas Cedex, France; 5 Architecture et Fonction des Macromolécules Biologiques, UMR6098, Centre National de la Recherche Scientifique, Universités Aix-Marseille I & II, Marseille, France; Wageningen University, Netherlands

## Abstract

The human intestine hosts a complex bacterial community that plays a major role in nutrition and in maintaining human health. A functional metagenomic approach was used to explore the prebiotic breakdown potential of human gut bacteria, including non-cultivated ones. Two metagenomic libraries, constructed from ileum mucosa and fecal microbiota, were screened for hydrolytic activities on the prebiotic carbohydrates inulin, fructo-oligosaccharides, xylo-oligosaccharides, galacto-oligosaccharides and lactulose. The DNA inserts of 17 clones, selected from the 167 hits that were identified, were pyrosequenced in-depth, yielding in total 407, 420 bp of metagenomic DNA. From these sequences, we discovered novel prebiotic degradation pathways containing carbohydrate transporters and hydrolysing enzymes, for which we provided the first experimental proof of function. Twenty of these proteins are encoded by genes that are also present in the gut metagenome of at least 100 subjects, whatever are their ages or their geographical origin. The sequence taxonomic assignment indicated that still unknown bacteria, for which neither culture conditions nor genome sequence are available, possess the enzymatic machinery to hydrolyse the prebiotic carbohydrates tested. The results expand the vision on how prebiotics are metabolized along the intestine, and open new perspectives for the design of functional foods.

## Introduction

The human gut hosts a complex microorganism community that is dominated by bacterial phylotypes belonging to Firmicutes, Bacteroidetes and Actinobacteria. The bacterial density increases along the digestive tract to reach 10^7^ and 10^11^ bacteria mg^−1^ of intestinal content in the distal parts of the small intestine (ileum) and of the large intestine (colon), respectively [1]–[4]. Collectively, gut bacterial genomes contain 150-fold more genes than the human genome equivalent [5], [6], [7], and code for many metabolic functions that make the gut microbiota a “virtual organ” which provides the host with missing metabolic capabilities that are crucial for maintaining its health and welfare [8], [9]. In particular, a huge diversity of enzymes involved in carbohydrate transport and metabolism are encoded by the gut metagenome, as revealed by Gill et al. [10]. Indeed, the intestinal microbiota contributes to host nutrition by harvesting energy from dietary fibers, these carbohydrates that are not digested in the upper part of the digestive tract because the human genome does not encode adequate carbohydrate active enzymes (CAZymes) [11]. Fibers are fermented by gut bacteria to produce organic acids (e.g., lactate, pyruvate, succinate), gases (e.g., H_2_, H_2_S, CO_2_ and CH_4_), and also short chain fatty acids (SCFA) (e.g., acetate, propionate and butyrate), these compounds contributing to the metabolic balance of the host [12]. Non-digestible carbohydrates include the natural constituents of vegetables, cereals, leguminous seeds and fruits, but also prebiotic oligosaccharides and polysaccharides [13]. Prebiotics are defined as food ingredients that fit to the three following criteria: 1) resistance to gastric acidity, to hydrolysis by mammalian enzymes, and to gastrointestinal absorption; 2) fermentation by intestinal microbiota; and 3) selective stimulation of the growth and/or activity of those intestinal bacteria that contribute to health and well-being [14], in particular bifidobacteria. For this last reason, the world market for prebiotics has grown rapidly in the last three decades [15], focusing on the production of compounds with established prebiotic effects (inulin, fructo-oligosaccharides, galacto-oligosaccharides and lactulose), as well as development and commercialization of other dietary carbohydrates, like resistant starch [16], xylo-oligosaccharides [17], gluco-oligosaccharides [18], [19], polydextrose [20], lactosucrose [21], pectin-derived [22] or soybean oligosaccharides [23].

However, the health benefits of prebiotics are controversial. Indeed, the causal relationship between increasing the number of bifidobacteria in the intestine and improving health status has not been established [24]. There is thus an urgent need to re-evaluate the impact of prebiotics on the intestinal microbiota as a whole, and not only on few cultivated *Bifidobacterium* and *Lactobacillus* species of whose growth is stimulated by prebiotics [25], [26], but which do not represent the ecosystem complexity. Several studies showed that other genera can be stimulated by prebiotics, directly or through cross-feeding of prebiotic breakdown products, such as *Bacteroides* [27], [28], *Faecalibacterium* [29], *Roseburia*, *Ruminococcus* and *Eubacterium* [30]–[32].

However, very little data are available regarding the enzymes that are directly and specifically involved in prebiotic metabolism by gut bacteria. Only a few studies based on sequence functional annotation [33], [34], transcriptomics [35]–[37], proteomics [38] or recombinant enzyme expression and *in vitro* characterization [26], [39]–[42] reported on the identification of genes and proteins involved in inulin, fructo-, xylo- and galacto-oligosaccharide metabolism by some cultivated *Bifidobacterium*, *Lactobacillus Roseburia*, *Faecalibacterium* and *Bacteroides* strains. Nevertheless, these strains constitute only a minor fraction of the gut microbial diversity.

In this paper, we present a screening-based functional metagenomics study to identify genes encoding key prebiotic degrading enzymes from both cultivated and non-cultivated gut bacteria. Both the ileum mucosa and fecal microbiota were targeted, to highlight the specific potential of prebiotic metabolization in two gut compartments and locations, which, to our knowledge, has never been studied. Our strategy was first based on the design of functional screens to guide the in-depth sequencing of metagenomic fragments encoding enzymes that hydrolyse five compounds sold as prebiotics for human consumption. Then, the occurrence in the human gut metagenome of the fished genes, obtained from two individuals, was studied in the light of the latest metagenomic data released from the Human Microbiome Project, to identify the genes that are shared by numerous individuals, whatever are their age or their geographical origin [43]. The results provided new insights on metabolism of inulin, fructo-oligosaccharides, lactulose, galacto-oligosaccharides, and xylo-oligosaccharides.

## Results and Discussion

### Screening the human gut metagenome for prebiotic-degrading activities

Twenty thousand clones of each of the two *E. coli* fosmid libraries constructed from fecal and ileum mucosa microbiota (named F and I libraries respectively), covering in total 1.4 Gb of metagenomic sequence, were screened for clone ability to degrade prebiotic compounds of various structures (Table 1). *E. coli* was chosen as the recombinant host because it was previously shown to allow expression of genes belonging to bacteria that are distantly related from a taxonomical point of view, like Bifidobacteria [44].

**Table 1:** Structure of the carbohydrate compounds used for primary and secondary screening and number of hit clones isolated from the fecal (F) and the ileum mucosa (I) libraries.

compounds	Screening steps^e^	Structure	Number of hit clones (Hit yield)	
			F library	I library
XOS^a^	1 and 2	Xyl-β(1,4)-[Xyl]n (1≤n≤7)	4 (0.20‰)	27 (1.35‰)
FOS^a^	1 and 2	Glc-α(1,2)-[β(1,2)-Fru]n (1≤n≤4)	7 (0.35‰)^b^	8 (0.4‰)^b^
Inulin	2	Glc-α(1,2)-[β(1,2)-Fru]n (10≤n)	7 (0.35‰)	8 (0.4‰)
AZCL-galactan	1	[β(1,4)-Gal]n (n≈100)	107 (0.79‰)	NT^d^
GOS^a^	2	[β(1,4)-Gal]n-Gal-β(1,4)-Glc (0≤n≤14)	107 (0.79‰)^c^	11 (0.55‰)^c^
Lactulose	1 and 2	Gal-β*(*1,4)-Fru	NT^d^	14 (0.7‰)

a XOS, xylo-oligosaccharides; FOS, fructo-oligosaccharides; GOS, galacto-oligosaccharides.

b Clones positive on fructo-oligosaccharides were further assayed for inulin degrading activity.

c Clones positive on lactulose (I library) and those identified by screening the entire F library (136, 000 clones) on AZCL-galactan were further assayed on galacto-oligosaccharides.

d NT: non tested.

e Screening steps: 1, high-throughput primary screen; 2, HPAEC-PAD based secondary screen.

First, a high-throughput primary search for clones growing on xylo-oligosaccharides, lactulose and fructo-oligosaccharides was performed using solid selective media containing the oligosaccharides as sole carbon source. Because the expression host EPI100 *E. coli* strain used to construct the library was unable to metabolize galactose, another strategy presented below has been applied to search for galacto-oligosaccharide degrading clones. The 120, 000 growth assays that were carried out allowed the identification of 11 and 49 hit clones from the F and I libraries, respectively. Hit yields varied between 0.2 and 1.35‰, depending on the library and on the oligosaccharide structure (Table 1).

In a second step, we validated and discriminated the prebiotic oligosaccharide hydrolysis ability of selected clones. For this purpose we performed HPAEC-PAD analysis of the products obtained after incubation of the cytoplasmic extracts with the prebiotic compound. All the clones growing on media containing xylo-oligosaccharides, fructo-oligosaccharides and lactulose were able to release mono-saccharides from these substrates (Table 2, Figure S1). In addition, this analysis showed that the clones selected by using fructo-oligosaccharides were also able to degrade inulin. Regarding the search for galacto-oligosaccharide degrading clones, the 14 I library clones found on lactulose were tested for galacto-oligosaccharide hydrolysis. Eleven clones were able to degrade oligosaccharides that were present in the commercial galacto-oligosaccharide preparation. The 107 clones which were identified in a previous study by screening 136, 000 clones from the F library for AZCL-galactan breakdown [44] were tested for galacto-oligosaccharide hydrolysis. All of them were able to degrade galacto-oligosaccharides (Table 1). Depending on the HPAEC-PAD profiles of their prebiotic degradation products (amount and polymerisation degree of the hydrolysis products) the hit clones were classified into 19 clusters. The 17 clones that were representative of clusters containing the most efficient clones were chosen for subsequent pyrosequencing of metagenomic DNA insert (Table 2).

**Table 2:** Clone classification according to the amount and degree of polymerisation of prebiotic hydrolysis products, determined by HPAEC-PAD analysis.

Origin of the metagenomic libary	Prebiotics	Cluster	Hydrolysis products	Number of positive clones	Identifiers of sequenced clones
Feces	FOS	1	Glc, Fru	4	F1-F15
		2	Glc, Fru, Glc-α(1,2)-Fru	2	F2-F16
		3	Low amounts of Fru, Glc-α(1,2)-[β(1,2)-Fru]n (1≤n≤3)	1	–
	Inulin	1	Glc, Fru, Glc-α(1,2)-[β(1,2)-Fru]n (n≤10)	4	F2-F15-F16
		2	Low amounts of Glc, Fru and Glc-α(1,2)-[β(1,2)-Fru]n (n≤10)	2	–
		3	Glc, Fru	1	F1
	XOS	1	Xyl	3	F3-F5-F17
		2	Xyl and Xyl-β(1,4)-Xyl	1	F4
	GOS	1	Gal, Glc	74	F6
		2	[β(1,4)-Gal]m (4≤m≤7), [β(1,4)-Gal]n-Gal-β(1,4)-Glc (2≤n≤5)	33	–
Ileum mucosa	FOS	1	Glc, Fru	6	I10
		2	Glc, Fru, Glc-α(1,2)-[β(1,2)-Fru]n (2≤n≤4)	2	I9
	XOS	1	Xyl	18	I7
		2	Xyl, Xyl-β(1,4)-Xyl	8	I8
		3	Low amount of Xyl	1	–
	Lactulose	1	Gal, Fru	12	I11-I12-I13-I14
		2	Low amount of Gal and Fru	2	–
	GOS	1	Gal, Glc, [β(1,4)-Gal]n-Gal-β(1,4)-Glc (1≤n≤14)	9	I11-I14
		2	Gal, Glc, [β(1,4)-Gal]n-Gal-β(1,4)-Glc (1≤n≤3)	2	I13

This study provides experimental evidence of the prebiotic degrading potential of non-cultivated gut bacteria, in particular those from ileum. This hydrolysis ability correlates with sequence-based metagenomic results which revealed that functions related to uptake and metabolism of relatively simple carbohydrates (mono- and di-saccharides) are well represented in the small intestine microbiota (6, 7). However, it also proves that ileum bacteria are also able to breakdown longer oligosaccharides (for instance fructo-oligosaccharides of polymerisation degree 5 or higher, like those present in inulin). Numerous prebiotics contain the same glycoside residues and the same glycosidic linkages as plant cell wall polysaccharides (cellulose, hemicellulose, pectin, galactan) or polysaccharides involved in plant energy storage (starch and inulin). Indeed, they are industrially produced by partial hydrolysis of these polysaccharides that also constitute natural dietary fibres and the main carbon sources for gut colonizing bacteria. Unlike the other prebiotics tested in this study, lactulose is not produced from plants; this is an artificial oligosaccharide that is chemically or enzymatically synthesized from lactose [45]. Lactulose is a structural isomer of lactose, a disaccharide that is present in most mammals' milk, including human [46] and bovine milks [47], and of galacto-disaccharides that derives from the enzymatic hydrolysis of the plant cell wall polysaccharide galactan. We thus propose that at least a part of the metabolic pathways of the natural dietary components lactose and galacto-oligosaccharides of gut bacteria is also responsible for the breakdown of the synthetic prebiotic lactulose.

### Identification of gene clusters assigned to prebiotic catabolism

Pyrosequencing of metagenomic DNA from the 17 selected clones was performed with a high sequencing depth (between 45X and 171X), ensuring reliable sequence assembly. For each clone, one contig sizing between 13, 000 and 39, 000 bp was obtained, except for clones 9 and 10 which generated two contigs each. Partial or complete sequence redundancy was observed for 4 clones from the I library and 6 clones from the F library, respectively (Table 3). As most of these redundant sequences were not assignable from a taxonomic point of view (based on sequence homology analysis with sequenced genomes), it was impossible to determine if their bacterial origin corresponds to over-represented species in the samples used for library construction.

**Table 3:** Sequence analysis of the metagenomic inserts isolated in this study: taxonomical and functional assignation, and gene occurrence in the human gut metagenome.

Sequenced Clone	Contig name and size	ORF number	Taxonomic assignation	Substrate	CAZy family	Number of ORFs assigned to G COG	Occurrence analysis d
F1	1^a^ (33, 125bp)	23	Clostridiales^b^	FOS & Inulin	GH32^c^	3	21/8
F2	2^a^ (37, 022bp)	33	*Bifidobacterium longum*	FOS & Inulin	GH32^c^	2	32/27
F3	3^a^ (35, 645bp)	26	Clostridiales^b^	XOS	GH67, GH2, GH3^c^	6	24/24
F4	4 (31, 477bp)	34	*Bifidobacterium adolescentis*	XOS	GH13, GH77, GH43^c^, GH8^c^, GH120^c^	5	27/17
F5	5 (39, 093bp)	27	*Bacteroides vulgatus*	XOS	GH43^c^, GH43^c^, GH43^c^, GH10^c^, GH16, CE9	9	24/24
F6	6 (37, 418bp)	29	*Bifidobacterium longum*	GOS	GH13-CBM48, GH2^c^, GH42^c^	6	25/25
I7	7^a^ (37, 821bp)	18	Bacteroidales^b^	XOS	GH115, GH10^c^, GH43^c^	5	17/0
I8	8^a^ (38, 518bp)	19	Bacteroidales^b^	XOS	GH35, GH67, GH115, GH10^c^, GH43^c^	7	17/0
I9	9^a^ (14, 714bp)	12	*Dorea longicatena*	FOS	GH32^c^	3	10/3
	9^b^ (13, 700bp)	11	*Clostridium nexile*	FOS	–	0	10/8
I10	10^a^ (11, 660bp)	10	*Eubacterium rectale*	FOS	GH32^c^	4	9/9
	10^b^ (12, 461bp)	15	*Eubacterium rectale*	FOS	GT2	1	11/10
I11	11^a^ (30, 347bp)	24	*Streptococcus* *thermophilus*	Lactulose & GOS	GT2-GT8, GH2^c^	5	19/1
I12	12 (13, 317bp)	12	Clostridiales^b^	Lactulose	GH42^c^, GH2^c^	6	10/9
I13	13 (32, 036bp)	30	*Faecalibacterium prausnitzii*	Lactulose & GOS	GH2^c^, GH77, GT35	5	29/11
I14	14^a^ (26, 887bp)	22	*Streptococcus thermophilus*	Lactulose & GOS	CBM41-CBM48, GH13, GH13, GH2^c^	8	16/8

a Contig 7 is partially redundant with contig 8 (ORFs 1 to 14 of contig 7 are identical to ORFs 5 to 19 of contig 8), contig 11 is partially redundant with contig 14 (ORFs 9 to 12 and 18 to 24 of contig 11 are identical to ORFs 11 to 22 of contig 14). In addition, contig sequences of clones 15, 16 and 17 were totally covered by contig 1, 2 and 3 sequences, respectively.

b MEGAN based assignation.

c CAZy families known to degrade the glycosidic bonds contained in the prebiotic substrate.

d Results of BLASTN comparison of the predicted ORFs with the metagenomic data sets issued from fecal sampling of 3 cohorts of 163, 139 and 110 subjects. The first number corresponds to the number of ORFs that present a sequence identity ≥90% and an E-value  = 0 with a gene detected at least in one subject of the three cohort. The second number corresponds to the number of ORFs that present a sequence identity ≥90% and an E-value  = 0 with a gene detected at least in 20 subjects.

In total, 213, 780 bp (containing 172 ORFs) and 193, 640 bp (containing 158 ORFs) of metagenomic DNA were analysed for F and I libraries, respectively. On average, 26% of the detected ORFs were assigned to the G COG cluster, corresponding to proteins that are predicted to be involved in Carbohydrate Transport and Metabolism (Tables 3 and S1). The dominance of G COG cluster, which was not observed in the randomly sequenced human gut metagenome [5], [48], [49], reflects as previously described [44], the power of screening-based strategies to guide the sequencing of DNA fragments that are rich in genes of interest.

Among these 330 ORFs, 38 were annotated as CAZyme encoding genes. Thirty-three of these predicted CAZymes contained a glycoside-hydrolase (GH) module (Tables 3, S1 and S2), others being esterases and glycosyl-transferases, which are not involved in prebiotic breakdown.

**Figure 1:**
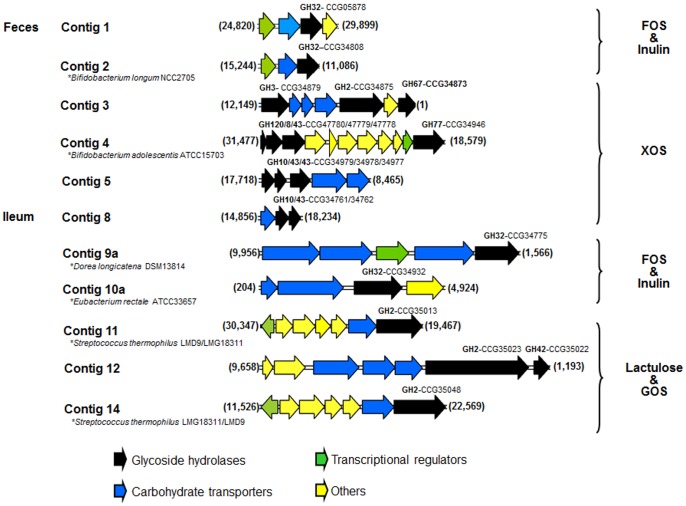
Operon-like structures encoding prebiotic degrading enzymes. The operon-like multigenic systems encoding proteins involved in carbohydrate binding, metabolism and transport surrounded by putative transcriptional regulators are shown. Genes are represented by arrows. Under the operon-like structures are reported the strains for which perfect synteny was identified between part of their genome and the present metagenomic sequences.

Moreover, on 11 contigs, the putative GHs are encoded by operon-like multigenic systems encoding proteins involved in carbohydrate binding, metabolism and transport (belonging to the ABC transport family, to PTS systems or to SusD and SusC families), surrounded by putative transcriptional regulators (Figure 1, Table S1). Five of the discovered GHs share more than 90% sequence identity with enzymes from *Bifidobacterium* species, which were already shown to be involved in prebiotic breakdown: one GH32 (contig F2) involved in fructo-oligosaccharide degradation [50]–[52], three GH43, GH8 and GH120 (contig F4), synergistically involved in the metabolization of xylo-oligosaccharides [42], [53], [54], and the last two predicted enzymes assigned to families GH2 and GH42 (contig F6), involved in the hydrolysis of galacto-oligasaccharides [55]. For all the other pathways of carbohydrate transport and catabolism highlighted here (encoded by contigs F1, F3, F5, I7, I8, I9, I10, I11, I14, I12, and I13), the present screening results constitute the first biochemical evidence of their ability to participate to prebiotic metabolization. Moreover, these results contribute to indicate functions of putative CAZyme sequences belonging to families which are often multispecific, and which contain only between 3 (for GH2) and 11% (for GH32) of characterized enzymes.

### Which are the bacteria that possess these prebiotic catabolic machineries?

To get new insights into the relationships between bacteria taxonomy and their role in prebiotic catabolism, the origin of the metagenomic DNA sequences was predicted. All the contigs came from bacteria of which the genome has not yet been sequenced, as none of the contig sequences were perfectly identical to sequenced genomes. However, for the contigs F2, F4, F5, F6, I10, I11, I13 and I14, the high sequence similarity with reference genomes made it possible to identify at the species level the nearest homolog of the gut bacteria of from which came the metagenomic inserts (Table 3). A near perfect synteny was observed with the genomic sequences of *Bifidobacterium longum* NCC2705 (contigs F2 and F6), *Bifidobacterium adolescentis* ATCC 15703 (contig F4), *Eubacterium rectale* ATCC 33656 (contig I10a), *Streptococcus thermophilus* LMD9 and LMG 18311 (contigs I11 and I14) (Figure 1 and Table S1). Regarding the contig F5, synteny with *Bacteroides vulgatus* ATCC 8482 was interrupted in the region carrying the GHs proposed to be responsible for the screened activity. Synteny between contig I13 and the *Faecalibacterium prausnitzii* KLE1255 genome was also highly perturbed. The gut bacteria from which came the DNA inserts F2, F4, F5, F6, I10, I11, I13 and I14 are thus probably very near, from a taxonomical point of view, of strains which were already described as being probiotics, carbohydrate fermenters, or, as being able to metabolize xylo-, fructo-, galacto-oligosaccharides or inulin [22], [30], [32], [33], [55]–[60]. However, as mentioned upper, apart from those of *Bifidobacterium longum* NCC2705, *Bifidobacterium adolescentis* ATCC 15703 and *Streptococcus thermophilus* LMD9, the enzymes involved in prebiotic degradation were not experimentally identified to date. The present metagenomic sequences are thus highly useful to pinpoint the corresponding gene machinery in the genome of cultivated strains. This is of prime importance for the design of innovative synbiotics, these functional food mixtures containing both a probiotic strain and the prebiotic compound that it is able to metabolize.

Regarding this aspect, clone I13, which was able to degrade lactulose and galacto-oligosaccharides, is of particular interest. Indeed, a cluster containing 17 putative genes (ORFs CCG34912 to CCG34928) were annotated as being involved in cobalamin (B12 vitamin) synthesis (Table S1). This vitamin plays a key role in the brain and in the nervous system [61]. Its absorption occurs by receptor-mediated endocytosis in the terminal ileum [62]. Cobalamin was already shown to be synthesized by bacteria present in the distal gut, in particular *Lactobacillus reuteri* strains that are marketed as probiotics and commonly used in the food industry [63]. Here, we showed that i) an ileum colonizing strain possesses both the machineries for lactulose/galacto-oligosaccharides hydrolysis and for putative cobalamin synthesis, ii) proteins which would be involved in prebiotic degradation and in vitamin synthesis share more than 90% identity with proteins of *Faecalibacterium cf. prausnitzii KLE1255*. These results allow enlarging the panel of prebiotic compounds that could stimulate the growth of *Faecalibacterium prausnitzii* species that could beneficially impact health [29], [64]. In particular, it would be interesting to test both the ability of *Faecalibacterium cf. prausnitzii* KLE125*5* to produce vitamin B12 and to hydrolyse lactulose and galacto-oligosaccharides, to evaluate its interest as a probiotic strain in synbiotics.

No significant sequence homology with lactobacilli genomic sequences was found, even if these bacteria are reported, with bifidobacteria, to be the most common prebiotic targets. This is probably due to the lower abundance of Lactobacillus species in the human gut microbiota, compared to Bifidobacterium species [2], [5], and to the fact that the gut metagenome diversity was not sufficiently covered by 40, 000 clones of 30 to 40 kbp each to isolate such low abundant sequences. Therein, based on 16S rRNA gene sequencing results, no Lactobacillus bacteria was found in the microbiota sample which was used to construct the F library [44], while Bifidobacterium bacteria represented 1% of this sample. No Lactobacillus bacteria was found in the ileum mucosa sample as well, but in this case, PCR based 16S rRNA gene taxonomic characterization was based only on 96 sequences, which is insufficient to be statistically reliable. One should thus not exclude that either cloning [65] or expression bias [66] may also have prevented the finding of lactobacilli sequences in this study, resulting from the use of E. coli as expression host.

Finally, the metagenomic inserts of clones F1, F3, I7, I8, I9, I12 were impossible to assign accurately, as their sequences were too distant from any available sequenced genome. However, Megan analysis of sequence homologies using low stringent criteria revealed that 5 of these sequences probably belong to bacteria of Clostridiales and Bacteroidales orders (Table 3). Moreover, the clone I9 metagenomic insert generated 2 contigs that were impossible to assemble. The first of these contigs (contig I9a), that encoded for a fructo-oligosaccharide metabolic gene cluster and a putative transposase (Figure 1, Table S1), presents a perfect synteny with a part of the *Dorea longicatena* DSM 13814 genome, while the second one (contig I9b) was highly homolog to a part of *Clostridium nexile* DSM 1788 genome. The PCR amplification of one gene in each contig allowed us to confirm that these two contigs came from one single metagenomic insert of one isolated clone. *Dorea longicatena* is one of the dominant species in human gut [5], and the DSM 13814 strain was previously shown to grow on inulin and on fructo-oligosaccharides [67], but we found no report on its eventual beneficial effects on human health. This chimeric construct could be an illustration of the human gut metagenome plasticity, mediated through horizontal gene transfers (HGTs) between nearly or distantly related bacteria, as previously described [44], [68]. Overall, these results highlight that growth of still uncharacterized commensal bacteria, of which the beneficial or deleterious effects for human health are unknown, could be stimulated by prebiotic oligosaccharides.

### Occurrence in the human gut metagenome of genes assigned to prebiotic breakdown pathways

Finally, we compared our metagenomic sequences, obtained from our 2 different subjects, with three different gene catalogues obtained from the random sequencing of fecal metagenomes, sampled from 162 subjects of MetaHIT cohorts [5], [69]; 139 subjects of NIH-HMP cohort [70] and other 110 subjects from different cultures, ages and families [43]. Striking results were obtained. Indeed, 87% of the genes identified in this study were at least 90% identical to genes detected in the gut metagenomic sequences of at least one subject of these cohorts (62% of genes being 100% identical), and 53% were at least 90% identical to sequences obtained from at least 20 individuals (Tables 3 and S1).

Anyway, the 20 genes constituting the operon-like gene clusters encoding prebiotic degrading enzymes obtained from clones F1 (non assigned), F3 (non assigned), F5 (assigned to *Bacteroides vulgatus*) and I10 (assigned to *Eubacterium rectale*) had highest occurences, whatever the targeted cohort. Indeed, they all are at least 90% identical to genes found in at least 100 different individuals. These results indicate that the present function-driven metagenomics study allowed the discovery of some of the major prebiotic metabolization machineries along the human digestive tract.

### Concluding remarks

In this study, we were able, by using a functional metagenomics approach, to directly access key enzymes involved in prebiotic metabolism by gut bacteria, including non-cultivated ones. We provided the first experimental evidence of the prebiotic hydrolytic potential in the ileum, and showed that in this gut compartment, bacteria are not only able to breakdown short carbohydrates like di-saccharides, but also longer ones, thanks to both exo- and endo-acting glycoside-hydrolases (like for instance polysaccharide degraders classified in the GH10 family).

Our results are consistent with the known ability of bifidobacteria to hydrolyse prebiotics, and allowed to evidence the prebiotic metabolization potential of strains currently used as probiotics or assumed to have health-promoting properties, which could thus be used to design novel functional foods like symbiotics. However, we also showed the high occurrence in human gut of still unknown bacteria possessing the enzymatic machinery to breakdown these compounds. Their growth could thus also be selectively stimulated by diet supplementation with prebiotics, thus generating effects on human health that remain to be determined. Prebiotic compounds, that were previously thought to direct a very specific response on human gut microbiota, exhibit a broader spectrum of action regarding target species but also gastro intestinal tract location. However, metagenome profiling, comparative metatranscriptomics and metaproteomics studies would have to be performed to evaluate the involvement level of the proteins that were identified here in real physiological conditions, with and without diet supplementation with prebiotics.

## Materials and Methods

### Metagenomic library construction

Two different gut microbiota were sampled as previously described [44], [71], in the frame of a study registered as a clinical trial (http://clinicaltrials.gov/, Calibrated Diets and Human Intestinal Microflora NCT00639561) and carried out at the Center for Clinical Investigation (CCI) of Grenoble University Hospital (France). The first one was a fecal sample of a healthy 30 years old male, who followed a vegetarian and fish-eating diet for twelve years before sampling. The distal ileum sample was obtained from a 51 years old male patient undergoing colonoscopy and surgery for lower colon cancer suspicion, after he has been submitted to a cleansing preparation. A segment of 2 cm^2^ was obtained, immediately frozen and kept at −80°C until processing. Both individuals showed an average body mass index. They did not eat any functional food such as prebiotics or probiotics, nor did receive any antibiotics or other drugs during 6 months before sampling. The sampling protocols were approved by the local ethics committee “Comité de Protection des Personnes Sud Est V” (Ref 07-CHUG-21, composed of B.Habozit, J.L.Crakowski, J.Juge, J.Grunwald, E.Svhan and E.Fontaine) and informed written consent was obtained from the subjects before sampling.

Metagenomic DNA was extracted, and fragments ranging in size from 30 to 40 kb DNA were isolated and cloned into pCC1FOS fosmid (Epicentre Technologies). EPI100 *E. coli* cells were then transfected to obtain a 156, 000 clone library from the fecal sample [44], and a 20, 000 clone library from the ileum sample [71]. Recombinant clones were transferred to 384-well microtiter plates containing Luria-Bertani medium, supplemented with 12.5 mg.l^−1^ chloramphenicol and 8% glycerol. After 22 h of growth at 37°C without any agitation, the plates were stored at −80°C.

### Carbohydrate sources

Commercial prebiotic oligosaccharides and polysaccharides used for screening were the following (Table 1): xylo-oligosaccharides (Iro Taihe International, China), fructo-oligosaccharides (Actilight-FOS, Beghin Meiji, France), inulin (from dahlia tuber, Sigma, Germany), galacto-oligosaccharides (Vivinal-GOS, FrieslandFoods Domo, The Netherlands), lactulose (TEVA, France).

Xylo-oligosaccharide preparations were purified by preparative chromatography to remove xylose, as following: 250 ml of a 50% (w/v) carbohydrate solution was loaded in a preparative-column (bed volume 7.8l, diameter 8 cm, column length 100 cm) filled with an ionic exchange resin (Amberlite CR1320K). Oligosaccharides were eluted by using MilliQ H_2_O and the carbohydrate content of each fraction was analysed by high-performance anion-exchange chromatography coupled to pulsed amperometric detection (HPAEC-PAD) as described in the following section. Fractions containing carbohydrates of polymerisation degree ≥2 were pooled, and the resulting solution was freeze-dried with an SMH 15 freeze-dryer (Société Nouvelle Usifroid, France). The final purity of the oligosaccharide powder was determined by HPAEC-PAD.

### High throughput functional screening

Twenty thousand clones from each library were gridded onto 22×22 cm trays containing minimum solid medium (Na_2_HPO_4_ 6 g.l^−1^, KH_2_PO_4_ 3 g.l^−1^, NH_4_Cl 1 g.l^−1^, NaCl 0.5 g.l^−1^, MgSO_4_ 0.12 g.l^−1^, CaCl_2_·2H_2_O 0.015 g.l^−1^, FeSO_4_·7H_2_O 0.0042 g.l^−1^, thiamine hydrochloride 0.0005 g.l^−1^, leucine 0.04 g.l^−1^, agar type E 15 g.l^−1^ and chloramphenicol 12.5 mg.l^−1^) supplemented with prebiotic oligosaccharides as sole carbon source, using a QPixII colony picker (Genetix), with a density of 2, 304 clones per tray. Oligosaccharides were sterilized by membrane filtration, using 0.20 µm Minisart filters (Sartorius Stedim Biotech GmbH, Germany) and added aseptically to the medium. The final concentration of carbohydrates was 0.5% (w/v) for xylo-oligosaccharides and lactulose, and 1% (w/v) for fructo-oligosaccharides. The trays were incubated at 37°C between 1 and 3 weeks, depending on the time necessary to visualize the growth of hit clones. It was checked that the EPI100 strain harboring the empty pCC1FOS vector is unable to grow on these selective media.

The hit clones were then streaked on agar LB medium supplemented with chloramphenicol 12.5 mgl^−1^, and the resulting isolated clones were stored at −80°C in liquid LB medium supplemented with chloramphenicol 12.5 mg.l^−1^ and glycerol 15% (w/v).

### Secondary screening

The prebiotic hydrolytic activities of the hit clones isolated from primary screening were further examined by HPAEC-PAD. The clones were grown at 37°C in 5 ml LB medium supplemented with chloramphenicol 12.5 mgl^−1^, with orbital shaking at 120 r.p.m. After 24 h, cells were harvested by centrifuging for 5 min at 5, 000 r.p.m., re-suspended in 1 ml potassium phosphate buffer 50 mM pH 7, containing 0.5 gl^−1^ lysozyme and incubated at 37°C for 1h. Cell lysis was completed with one freeze (−80°C) and thaw (30°C) cycle. Cell debris were centrifuged at 13, 200 r.p.m. for 10 min and the cytoplasmic extracts were filtered with a 0.20 μm Minisart RC4 syringe filter. Enzymatic reactions were carried out at 37°C by adding 0.2 ml of a 5% carbohydrate solution (w/v) to 0.8 ml of crude cytoplasmic extract. Reactions were stopped after 24 h by heating at 90°C for 5 min. Samples were diluted 200 times with MilliQ water and analysed by HPAEC-PAD on a Dionex ICS-3 000 system (Dionex Corp., Sunnyvale, CA) equipped with a CarboPac PA100 4×250 column connected to the corresponding guard column (Dionex).

The analyses were carried out at 30°C with a flow rate of 1 ml.min^−1^ with the following multistep gradient: 0–30 min (0–60% B), 30–32 min (60–90% B), 32–36 min (90–0% B) and 36–46 min (0% B). For analysing inulin hydrolysis products, samples were diluted twice in 1M NaOH, and the following multistep gradient was used: 0–15 min (0–30% B), 15–70 min (30–60% B), 70–125 min (60–90% B), 125–135 min (90% B), 135–150 min (90–0% B). Solvents were 150 mM NaOH (eluent A) and 150 mM NaOH, 500 mM CH_3_COONa (eluent B). We checked that the EPI100 *E.coli* host was unable to degrade the prebiotics tested in this study, as no prebiotic breakdown was detected by HPAEC-PAD, after having incubated during 24 h at 37°C the cytoplasmic and the total crude extracts of the EPI100 strain harboring the empty pCC1FOS vector with 5% of each prebiotic.

### Pyrosequencing, read assembly and ORF detection

Fosmid pyrosequencing was performed using a 454 GS FLX System (454 Life Science, CT) by the INRA Genomic Platform (Auzeville, France). Read assembly was performed using CAP3 software [72]. Contigs showing a sequence length <1, 000 bp and a sequencing depth <8 were removed. Remaining contigs were cleaned from the vector pCC1FOS sequence using Crossmatch (http://bozeman.mbt.washington.edu/phredphrapconsed.html). Open reading frames (ORF) of at least 20 amino acids were predicted using MetaGene [73]. Annotated contig sequences were deposited in European Nucleotide Archive under accession numbers: HE663537 and HE717006 to HE717020 (Table S2).

### ORF analysis

CAZyme encoding genes were identified by BLAST analysis of the predicted ORFs against the functional modules of glycoside hydrolases, polysaccharide lyases, carbohydrate esterases, carbohydrate-binding modules and glycosyltransferases included in the CAZy database (http://www.cazy.org) using a cut-off E-value of 7•10^−6^ followed by visual inspection and alignment with known CAZy families. Functions of other ORFs were inferred and manually annotated, based on BLASTX analysis against the NCBI non redundant and environmental database (E-value <10^−8^, identity >35%, query length coverage ≥50%). ORFs were assigned to clusters of orthologous groups of proteins (COGs) using RPS-BLAST analysis against the COG database (E-values ≤10^−8^). Finally, gene occurrence in the human gut microbiota was determined by TBLASTN comparisons of the predicted ORFs with the metagenomic data sets available, from fecal sampling of 162 subjects of the MetaHIT cohort (http://www.bork.embl.de/~arumugam/Qin_et_al_2010/http://www.bork.embl.de/Docu/Arumugam_et_al_2011/data/genes/; [5], [69]); of 139 U.S. subjects from the NIH-HMP cohort (http://www.hmpdacc.org/HMASM/; [70] and of the 110 subjects from different cultures, ages and families (http://metagenomics.anl.gov/projects/98; [43] (E-value  = 0, identity ≥90% and  = 100%).

Contig taxonomic assignment was based on ORF sequence similarity with sequenced genomes, using BLASTX analysis against the non-redundant NCBI database (E-value ≤10^−8^, identity ≥90%, query length coverage ≥50%). Contigs were assigned to a class, genus or species only if at least 50% of the ORFs were assigned to the same organism. Contigs containing ORFs assigned to different classes were not assigned. The most probable common ancestor of the organism from which the non-assignable contigs came from was retrieved using MEGAN v3.2.1, [74], based on BLASTX analysis against the non-redundant NCBI.

## Supporting Information

Figure S1 **HPAEC-PAD analysis of reaction products resulting from prebiotic hydrolysis.**
(DOC)

Table S1 Characteristics of metagenomic clones.
(PDF)

Table S2 Glycoside hydrolase families found in the study.
(DOC)
