# Supplementary material for: Functional Metagenomics Reveals Novel Pathways of Prebiotic Breakdown by Human Gut Bacteria
Source: PLoS One. 2013 Sep 16;8(9):e72766. doi: 10.1371/journal.pone.0072766 (PMC3774763; doi:10.1371/journal.pone.0072766)

**Figure S1** HPAEC-PAD analysis of reaction products resulting from prebiotic hydrolysis, after 24 h, by the 17 sequenced metagenomic clones. Chromatograms Y axis: nC (nano-Coulomb); X axis: retention time (min); DP: degree of polymerization

**Clone1-Fructo-oligosaccharides. Similar chromatograms were obtained for Clone 15.**


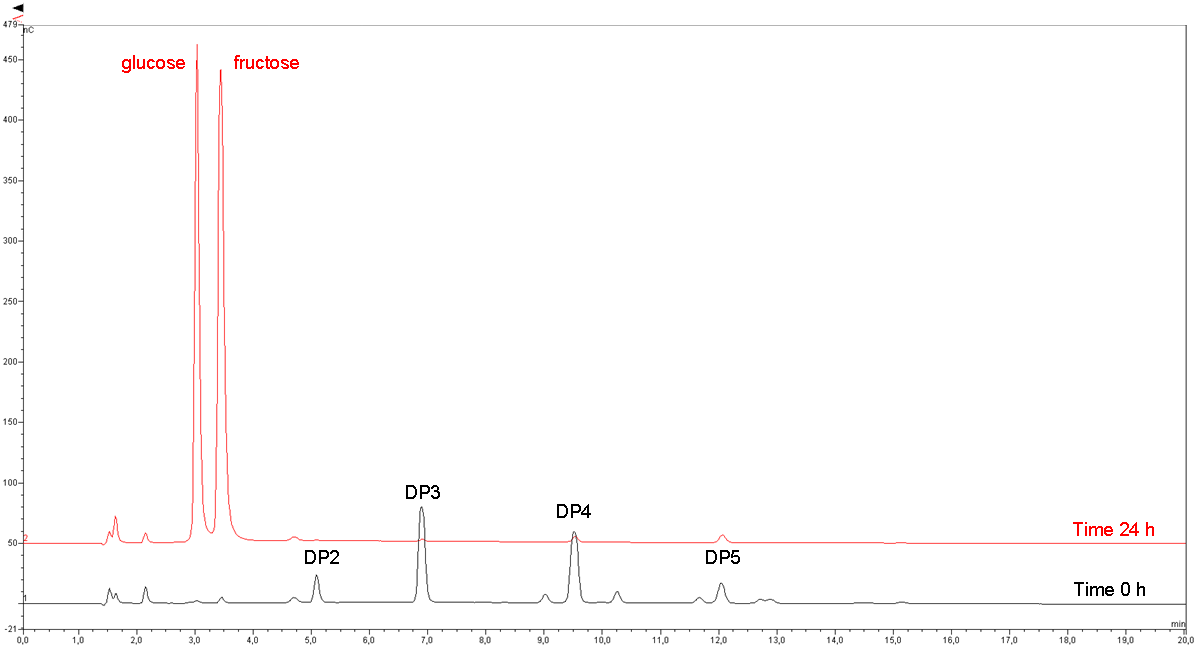


**Clone1-Inulin. Similar chromatograms were obtained for Clone 15.**


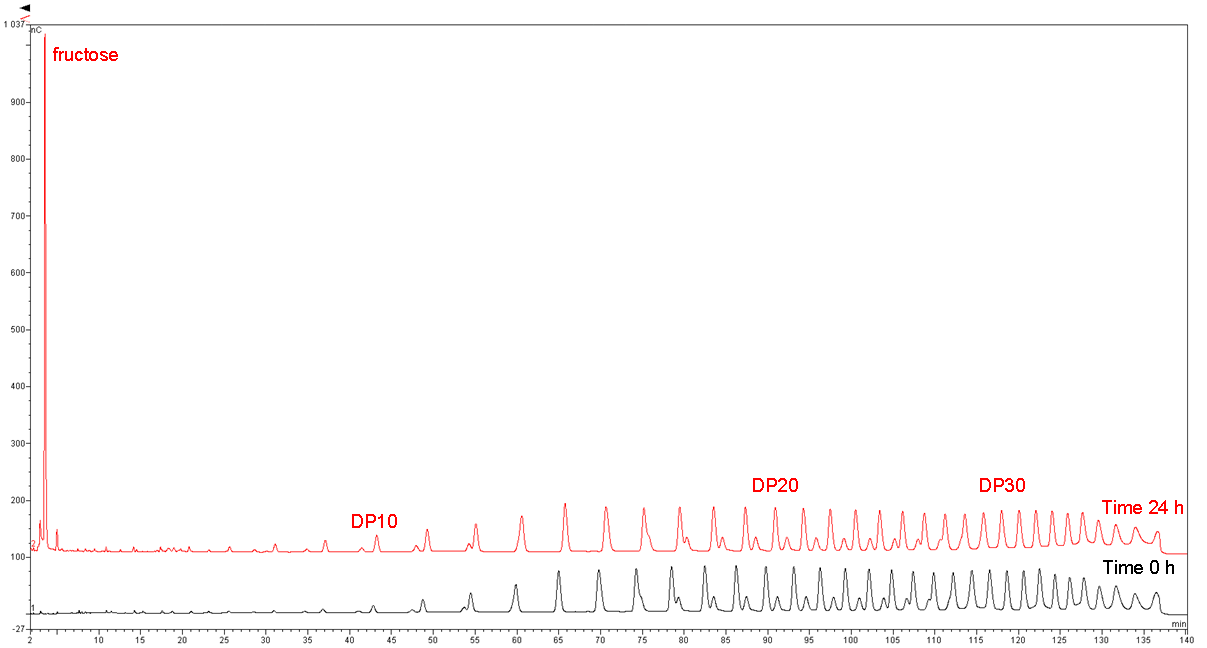


**Clone2-Fructo-oligosaccharides. Similar chromatograms were obtained for Clone 16.**


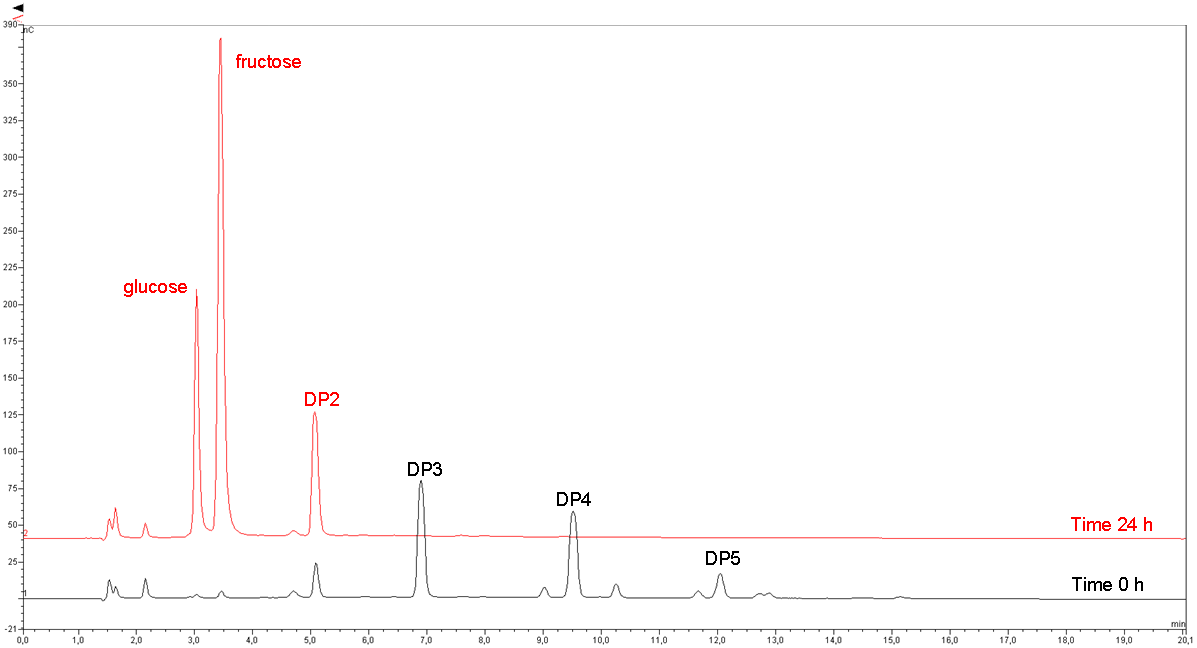


**Clone2-Inulin. Similar chromatograms were obtained for Clone 16.**


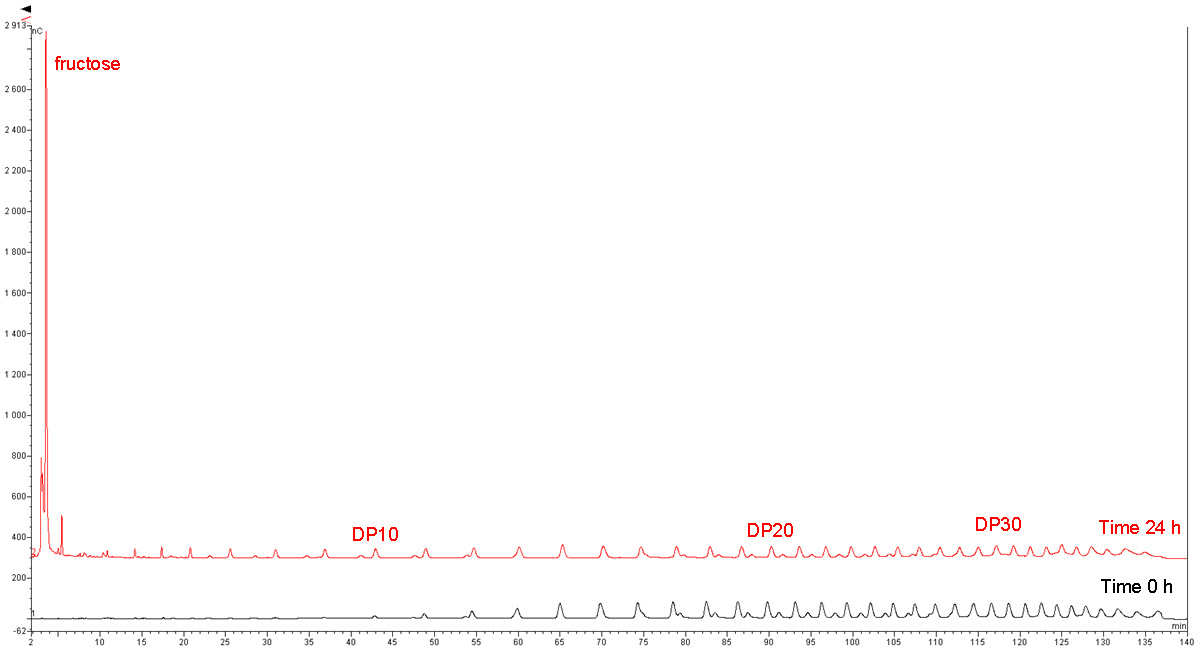


**Clone3-Xylo-oligosaccharides. Similar chromatograms were obtained for Clone 17.**


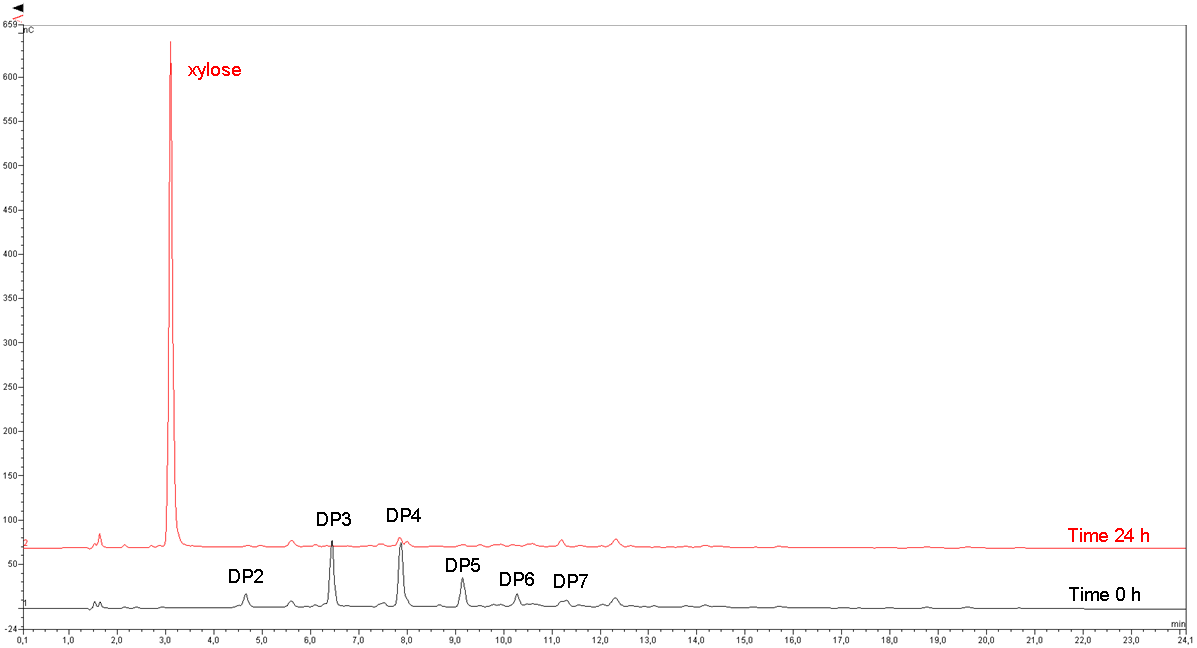


**Clone 4-Xylo-oligosaccharides**


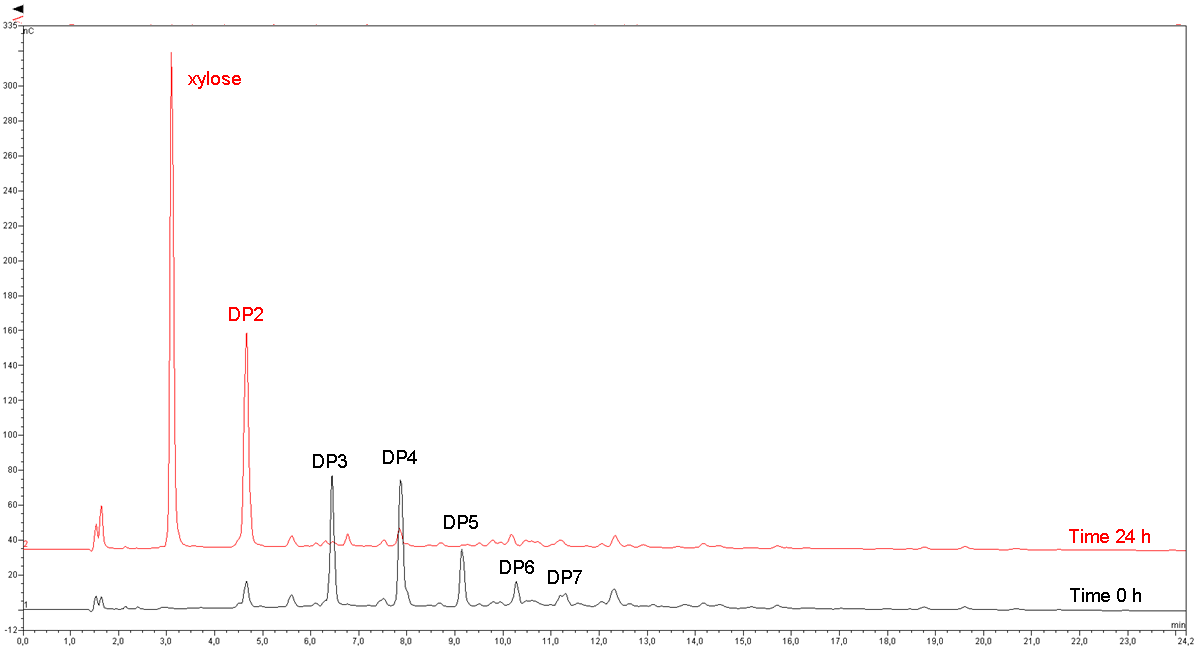


**Clone5-Xylo-oligosaccharides**


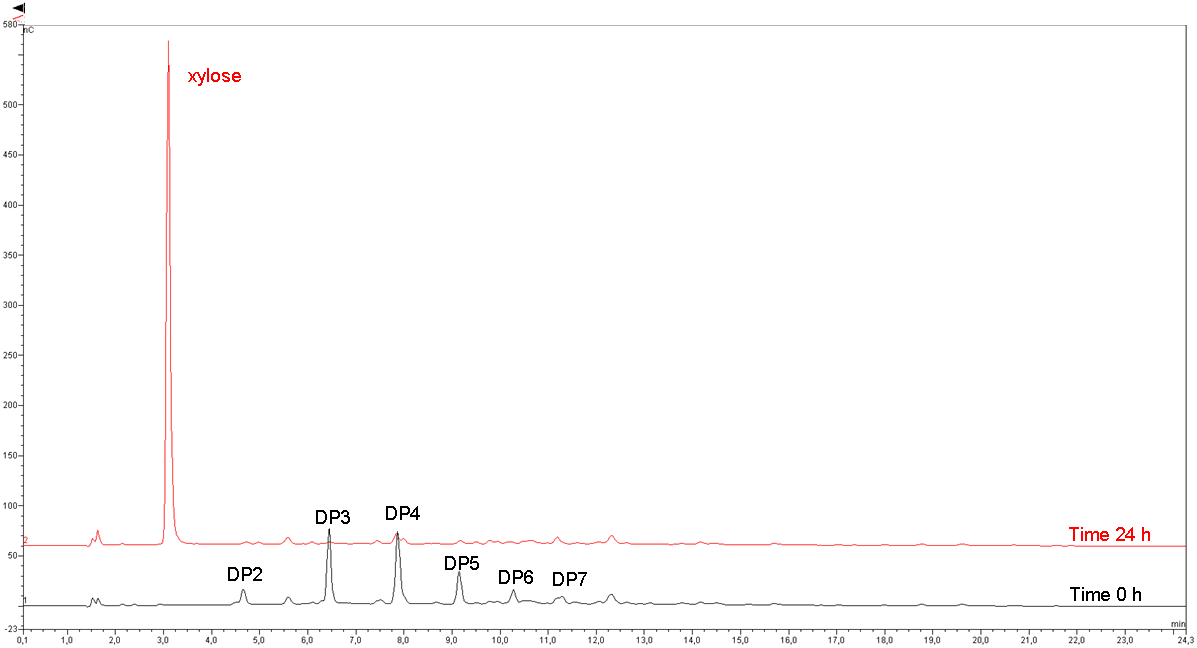


**Clone6-Galacto-oligosaccharides**


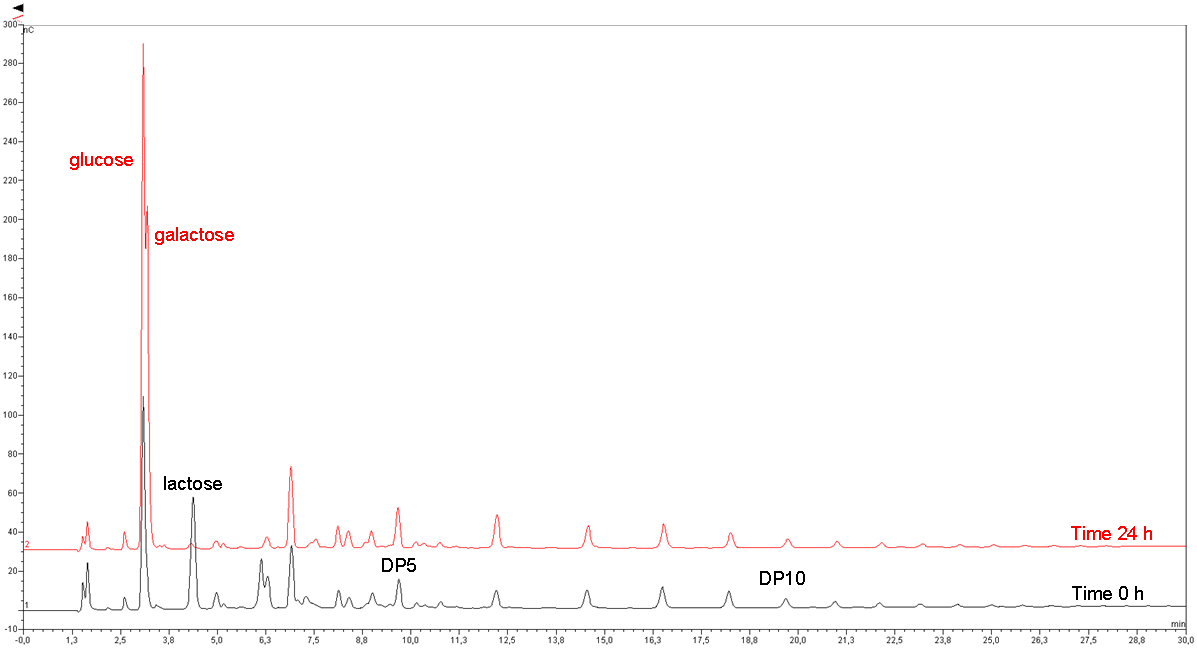


**DP5**

**DP10**

**Clone7-Xylo-oligosaccharides**


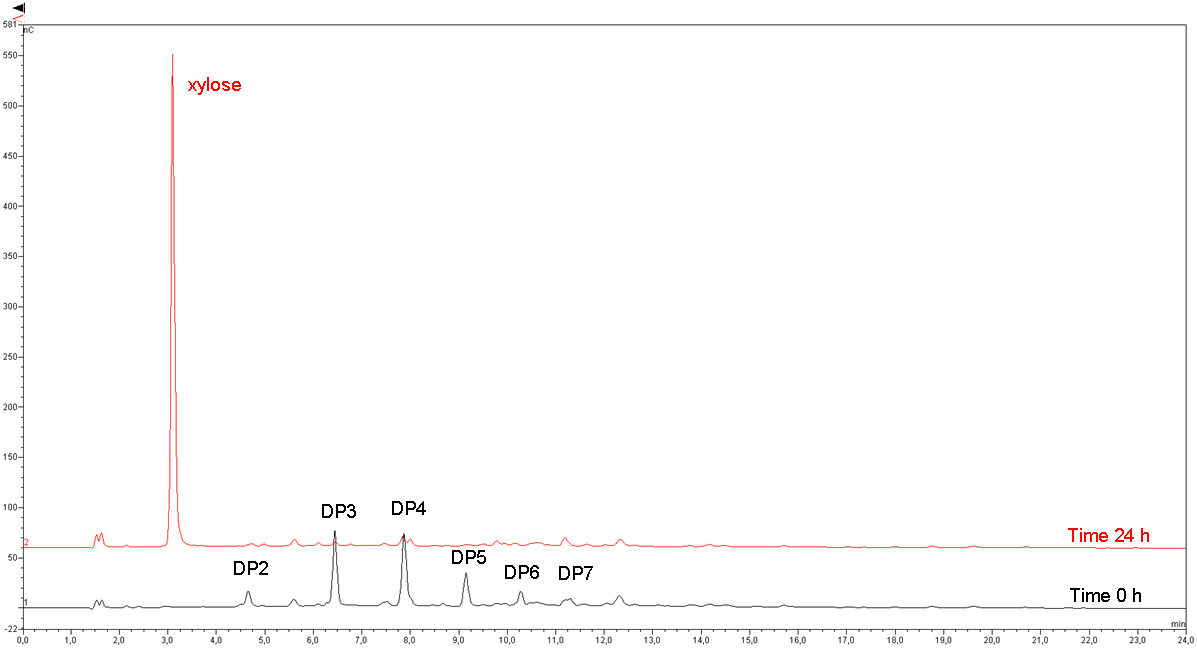


**Clone8-Xylo-oligosaccharides**


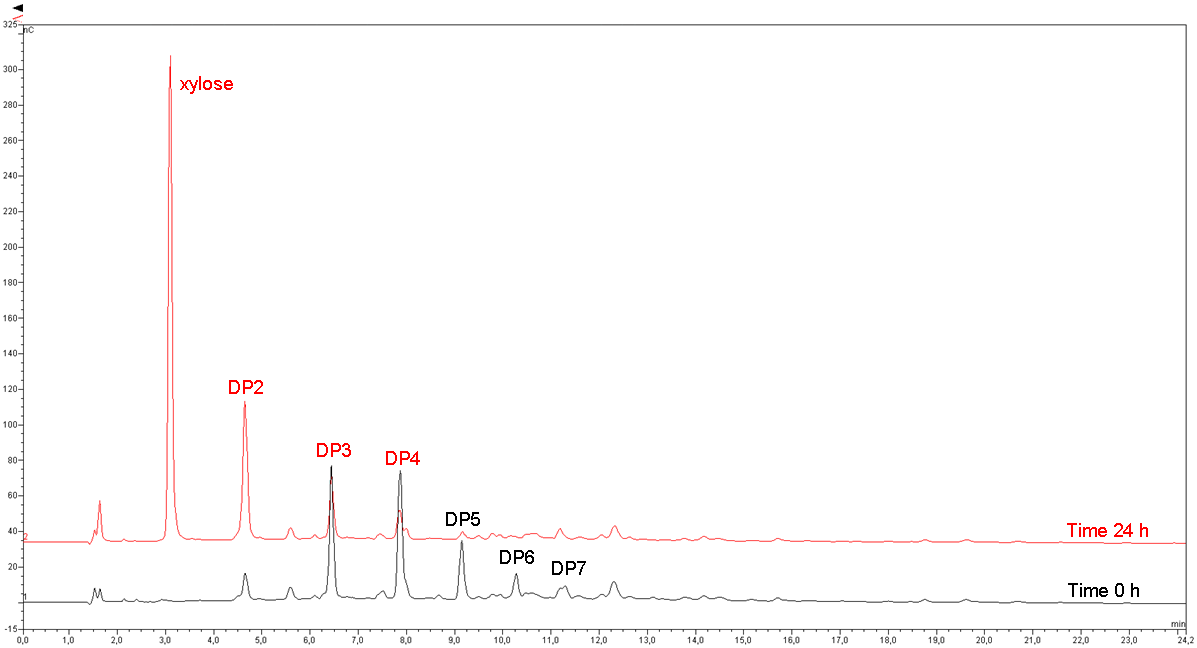


**Clone9-Fructo-oligosaccharides**


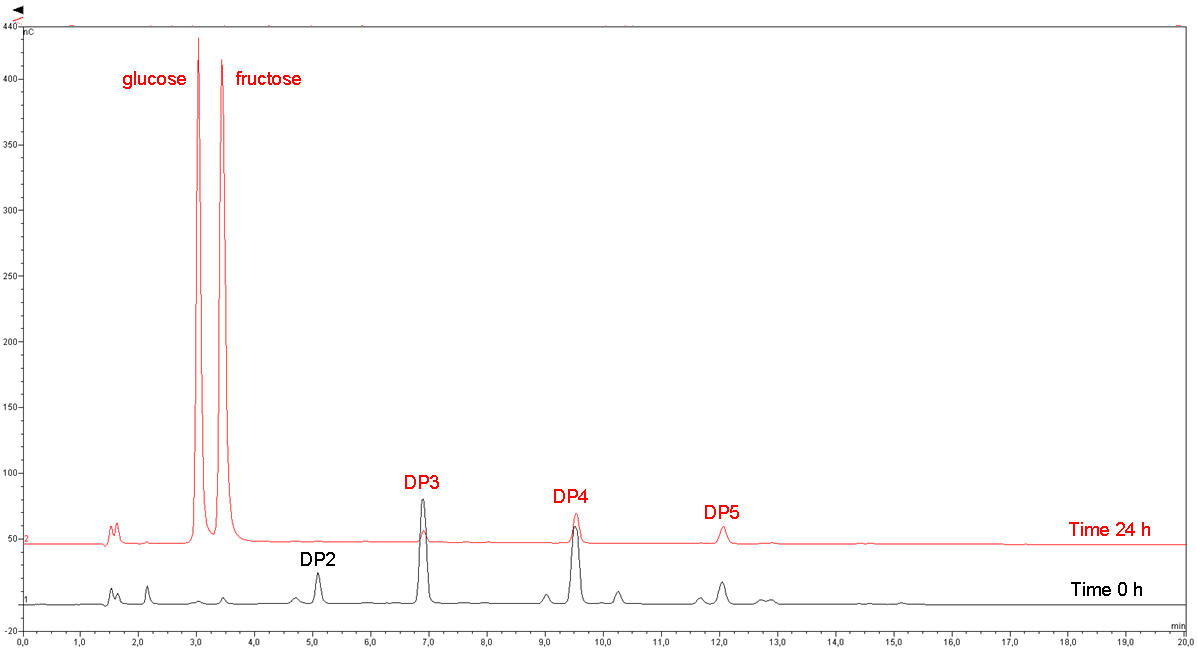


**Clone10-Fructo-oligosaccharides**


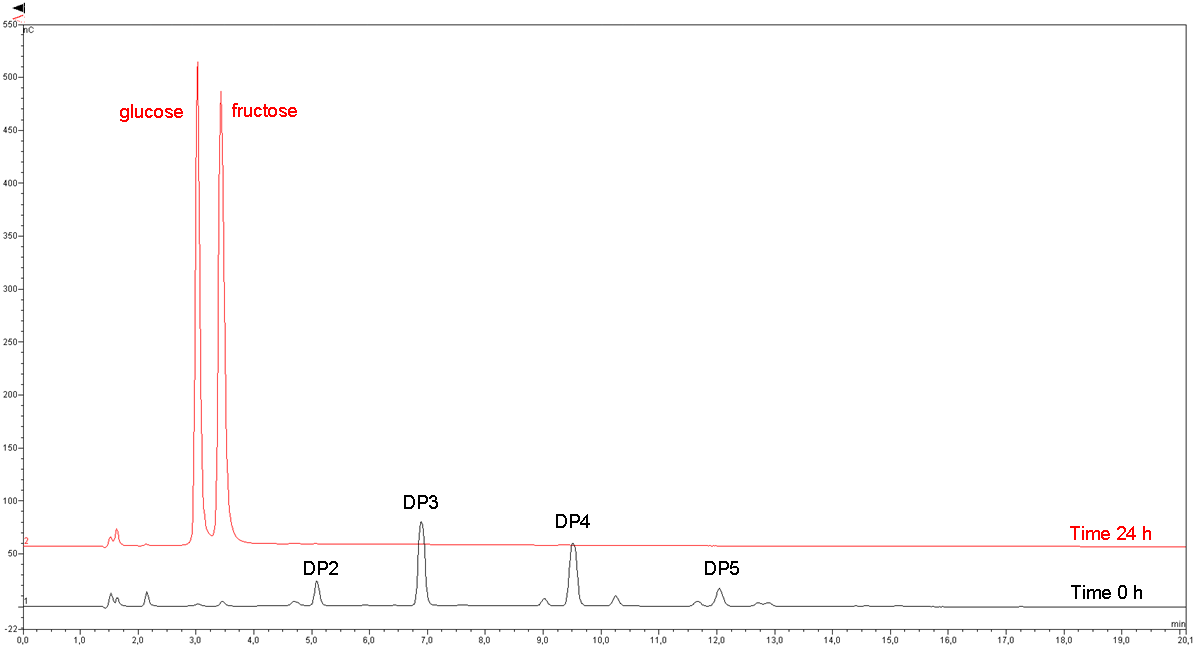


**Clone11-Lactulose**


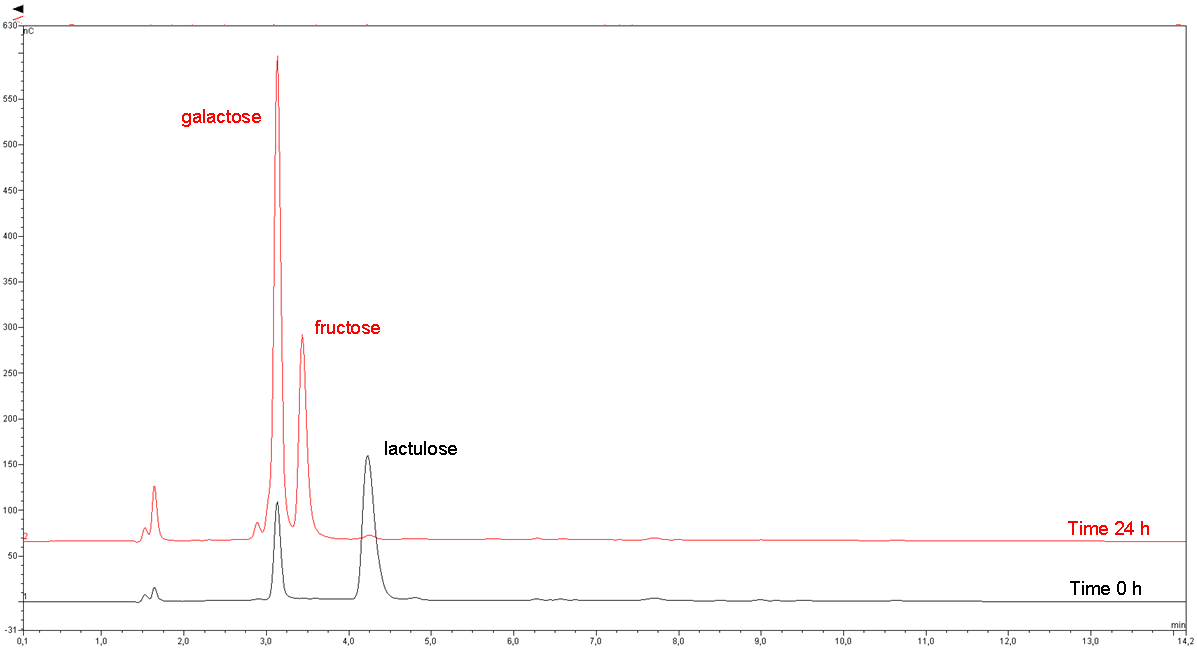


**Clone11-Galacto-oligosaccharides**


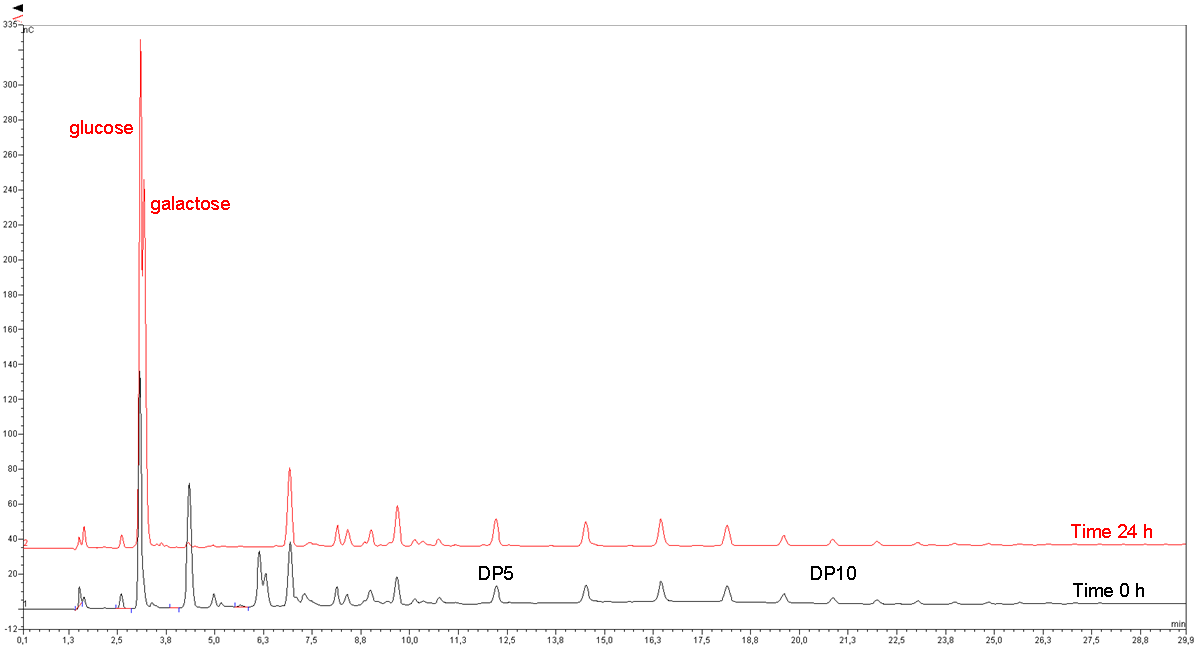


**Clone12-Lactulose**


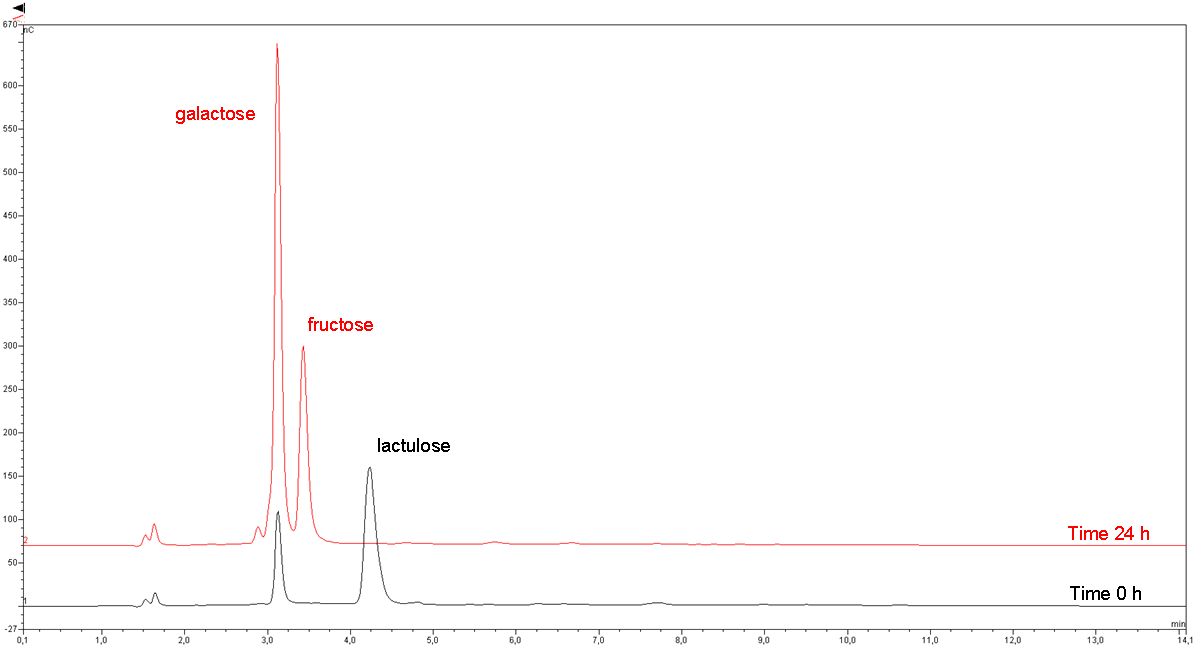


**Clone13-Lactulose**


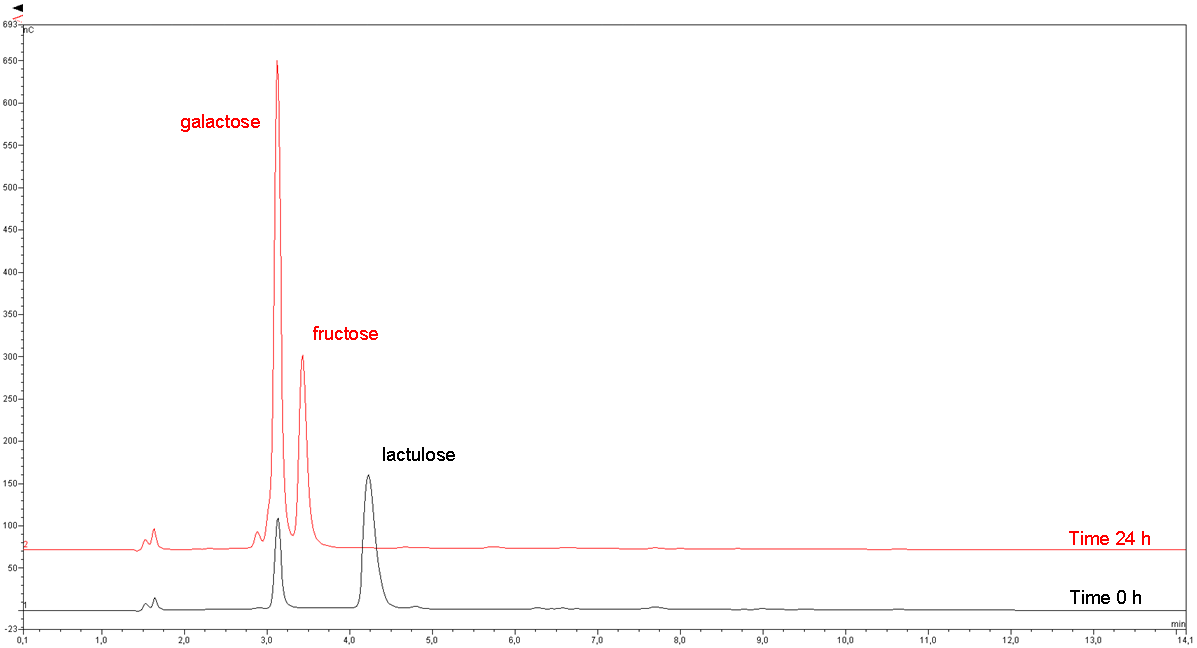


**Clone13-Galacto-oligosaccharides**


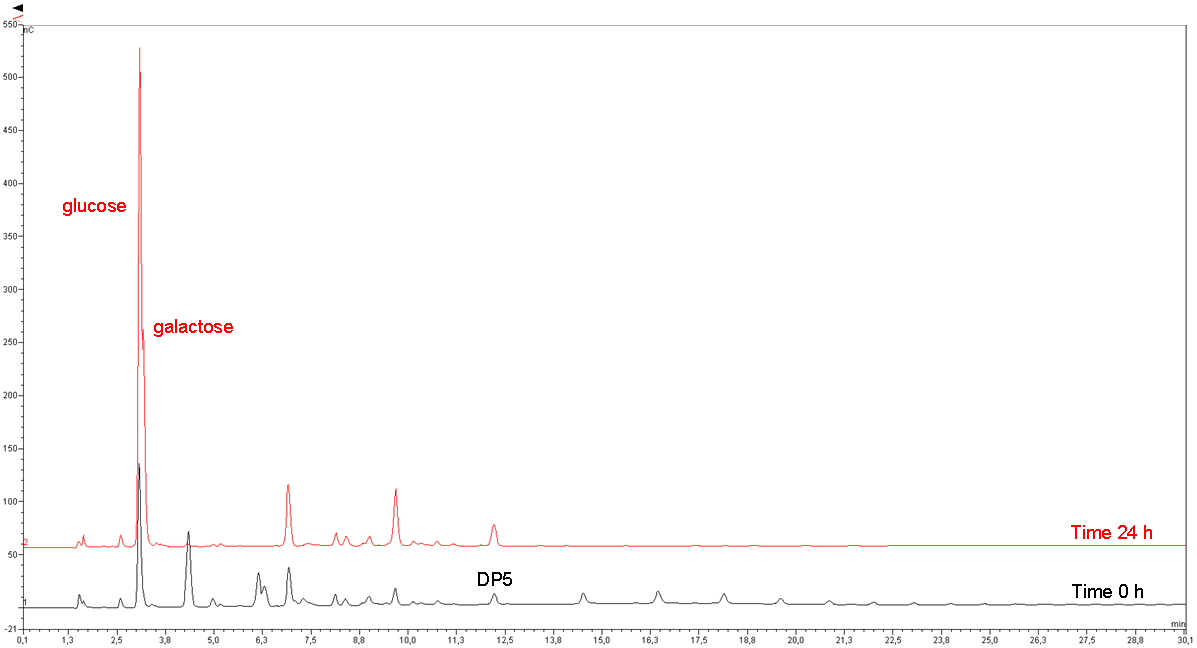


**Clone14-Lactulose**


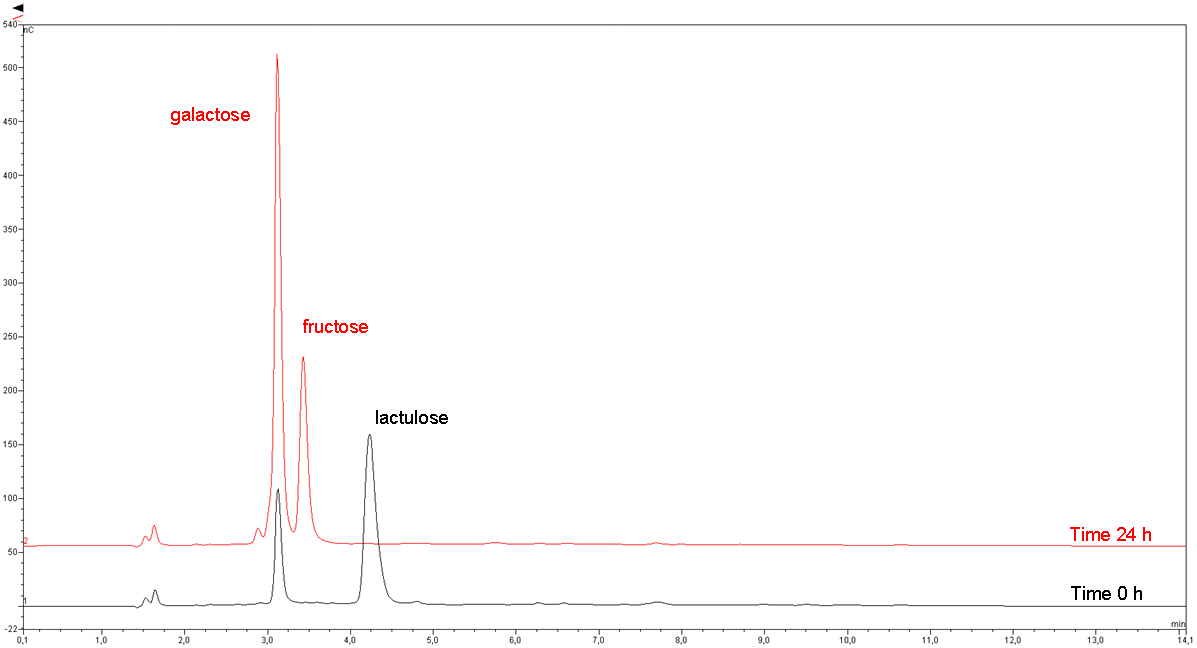


**Clone14-Galacto-oligosaccharides**


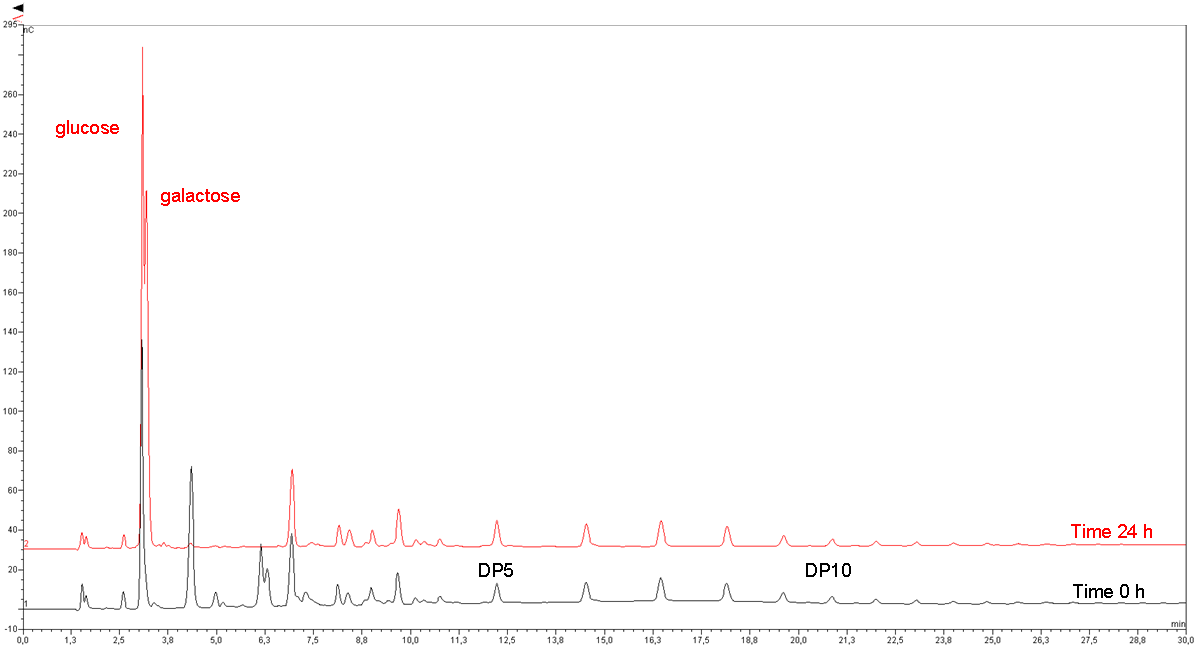

Supplement: Figure S1 — HPAEC-PAD analysis of reaction products resulting from prebiotic hydrolysis. (DOC) [file pone.0072766.s001.doc]
