# Supplementary material for: Functional Metagenomics Reveals Novel Pathways of Prebiotic Breakdown by Human Gut Bacteria
Source: PLoS One. 2013 Sep 16;8(9):e72766. doi: 10.1371/journal.pone.0072766 (PMC3774763; doi:10.1371/journal.pone.0072766)
Supplement: Table S1 — Characteristics of metagenomic clones. (PDF) [file pone.0072766.s002.pdf]

Clone identifier &substrate &Taxonomic assignment	Contig identifier & size(bp) &accession number of the contig sequence	ORF ID	N°ORF	strand	ORF functional assignment	higher % of identity to a characterized CAZyme of the same familly	COG assignment		prot. ID of best BLASTP hit	Bacterial origin of the best BLASTP hit	Occurrence in Metahit cohort (163 European subjects)		Occurrence in NIH - HMP cohort (139 USA subjects)		Occurrence in Age-Geo cohort (110 subjects)	
							Number	Letter			id≥90%	id=100%	id≥90%	id=100%	id≥90%	id=100%
					FECES											
F1 FOS Inulin Clostridiales*	F1 33125 bp HE663537	CCG05859	1	+	Growth inhibitor		COG2337	T			0	0	0	0	1	0
		CCG05860	2	+	Sitespecific recombinase XerD		COG4973	L		Faecalibacterium prausnitzii SL33]	47	0	55	0	7	0
		CCG05861	3	+	predicted protein		none	none			9	0	18	0	0	0
		CCG05862	4	+	putative TnpV protein		none	none			11	0	17	0	0	0
		CCG05863	5	+	predicted protein		none	none			0	0	0	0	0	0
		CCG05864	6	+	RelaxaseMobilisation nuclease domain		none	none			14	1	15	0	0	0
		CCG05865	7	+	putative transcriptional regulator		COG2865	K			12	0	19	0	0	0
		CCG05866	8	-	nucleaserelated domain protein		none	none			6	0	11	4	0	0
		CCG05867	9	-	conserved hypothetical protein		none	none			11	0	14	0	0	0
		CCG05868	10	-	putative DNA helicase		COG1205I	NF			9	0	12	0	1	0
		CCG05869	11	-	hypothetical protein CLOM621_06135		none	none			6	1	12	0	0	0
		CCG05870	12	-	TG mismatchspecific endonuclease		COG3727	L			5	0	9	0	0	0
		CCG05871	13	-	predicted protein		none	none			6	0	6	0	0	0
		CCG05872	14	-	predicted protein		none	none			6	0	8	0	0	0
		CCG05873	15	+	hypothetical protein		none	none			0	0	0	0	0	0
		CCG05874	16	+	prophage LambdaCh01 sitespecific recombinase phage integrase family		COG4973	L			23	4	37	5	1	0
		CCG05875	17	-	conserved hypothetical protein		none	none			62	2	46	0	3	0
		CCG05876	18	+	***transcriptional regulator LacI family***		COG1609	K			**56**	**1**	**48**	**0**	**7**	**0**
		CCG05877	19	+	**PTS system glucose subfamily IIA component**		COG1263	G			**79**	**3**	**63**	**4**	**5**	**0**
		CCG05878	20	+	**Glycoside hydrolase family 32**	41%	COG1621	G			**69**	**0**	**59**	**0**	**7**	**0**
		CCG05879	21	+	**putative fructokinase**		COG0524	G			**66**	**0**	**56**	**1**	**6**	**0**
		CCG05880	22	+	hypothetical protein CpapDRAFT_1802		none	none			6	0	2	2	0	0
		CCG05881	23	-	anaerobic ribonucleosidetriphosphate reductase		COG1328	F		Subdoligranulum variabile DSM 15176	55	0	62	14	6	1
F2 FOS Inulin *Bifidobacterium longum*	F2 37022 bp HE717010	CCG34796	1	+	Pyridoxine biosynthesis enzyme		none	none	NP_695325	Bifidobacterium longum NCC2705	24	8	6	3	42	9
		CCG34797	2	+	1deoxyDxylulose 5phosphate reductoisomerase		COG0743	I	NP_695326	Bifidobacterium longum NCC2705	18	3	5	3	45	6
		CCG34798	3	+	4hydroxy3methylbut2en1yl diphosphate synthase		COG0821	I	NP_695327	Bifidobacterium longum NCC2705	13	4	2	1	40	2
		CCG34799	4	+	Hydrolases of the alphabeta superfamily		none	none	NP_695328	Bifidobacterium longum NCC2705	23	0	9	3	37	13
		CCG34800	5	+	Recombinational DNA repair protein RecF pathway		COG1381	L	NP_695329	Bifidobacterium longum NCC2705	18	7	3	2	27	9
		CCG34801	6	+	Undecaprenyl pyrophosphate synthase		COG0020	I	NP_695330	Bifidobacterium longum NCC2705	14	1	5	4	46	10
		CCG34802	7	-	inosineuridine nucleoside Nribohydrolase		COG1957	F	NP_695331	Bifidobacterium longum NCC2705	17	0	4	0	41	0
		CCG34803	8	-	inosineuridine nucleoside Nribohydrolase		none	none	NP_695331	Bifidobacterium longum NCC2705	0	0	0	0	0	0
		CCG34804	9	-	ABCtype nitratesulfonatebicarbonate transport systems periplasmic components		COG0715	P	NP_695332	Bifidobacterium longum NCC2705	15	5	7	7	40	11
		CCG34805	10	-	ABCtype nitratesulfonatebicarbonate transport system permease component		COG0600	P	NP_695333	Bifidobacterium longum NCC2705	15	0	7	1	45	6
		CCG34806	11	-	**Glycoside hydrolase family 32**	99%	COG1621	G	NP_695334	Bifidobacterium longum NCC2705	**17**	**1**	**6**	**2**	**49**	**5**
		CCG34807	12	-	**sucrose transport protein**		none	none	NP_695335	Bifidobacterium longum NCC2705	**21**	**5**	**7**	**4**	**26**	**9**
		CCG34808	13	-	***LacI family transcriptional regulator***		COG1609	K	NP_695336	Bifidobacterium longum NCC2705	**17**	**2**	**9**	**5**	**49**	**6**
		CCG34809	14	-	serine protease inhibitor		COG4826	O	NP_695337	Bifidobacterium longum NCC2705	22	3	8	0	48	11
		CCG34810	15	-	hypothetical protein BLD_0127		none	none	NP_695338	Bifidobacterium longum NCC2705	10	9	5	5	16	4
		CCG34811	16	+	conserved hypothetical protein		none	none		Bifidobacterium longum subsp. infantis ATCC 55813	0	0	0	0	0	0
		CCG34812	17	-	putative permease		COG2271	G	NP_695340	Bifidobacterium longum NCC2705	25	1	6	2	42	11
		CCG34813	18	-	COG0011: Uncharacterized conserved protein		COG0011	S		Bifidobacterium longum DJO10A	9	4	3	2	30	5
		CCG34814	19	-	hydroxymethylpyrimidinephosphomethylpyrimidine kinase		COG0351	H	NP_695342	Bifidobacterium longum NCC2705	16	7	7	2	48	4
		CCG34815	20	+	Vesiclefusing ATPase		none	none		Bifidobacterium longum subsp. infantis ATCC 55813	0	0	0	0	0	0
		CCG34816	21	-	thiamine biosynthesis protein		COG0422	H	NP_695344	Bifidobacterium longum NCC2705	25	10	7	7	47	10
		CCG34817	22	-	hydroxyethylthiazole kinase		COG2145	H	NP_695345	Bifidobacterium longum NCC2705	15	2	3	2	47	10
		CCG34818	23	+	GlycyltRNA synthetase class II		COG0423	J	NP_695346	Bifidobacterium longum NCC2705	20	6	5	2	47	12
		CCG34819	24	+	tRNAdihydrouridine synthase		COG0042	J	NP_695347	Bifidobacterium longum NCC2705	15	5	5	3	47	11
		CCG34820	25	+	Cell division GTPase		COG0206	D	NP_695348	Bifidobacterium longum NCC2705	17	2	10	5	36	16
		CCG34821	26	+	cell division family protein		COG1799	S	NP_695349	Bifidobacterium longum NCC2705	9	2	5	1	38	0
		CCG34822	27	+	hemolysinlike protein		None	None	NP_695350	Bifidobacterium longum NCC2705	0	0	0	0	0	0
		CCG34823	28	+	probable cell division initiation protein		COG3264	M	NP_695351	Bifidobacterium longum NCC2705	18	7	10	2	44	7
		CCG34824	29	+	lipoprotein signal peptidase		COG0597	MU	NP_695352	Bifidobacterium longum NCC2705	19	3	4	0	40	3
		CCG34825	30	+	pseudouridine synthase RluA family		COG0564	J	NP_695353	Bifidobacterium longum NCC2705	18	5	6	5	41	13
		CCG34826	31	+	Predicted ATPase AAA superfamily		COG1373	R	NP_695354	Bifidobacterium longum NCC2705	12	6	1	0	11	4
		CCG34827	32	+	ribonuclease BN		COG1295	S	NP_695355	Bifidobacterium longum NCC2705	14	1	4	0	44	1
		CCG34828	33	+	DNA polymerase III alpha subunit		COG0587	L	NP_695356	Bifidobacterium longum NCC2705	24	0	4	0	43	6
F3 XOS Clostridiales*	F3 35645 bp HE717013	CCG34873	1	-	**glycoside hydrolase family 67**	55%	COG3661	G			**63**	**1**	**56**	**1**	**4**	**0**
		CCG34874	2	-	**cysteine desulfurase**		COG1104	E			**57**	**0**	**46**	**1**	**2**	**0**
		CCG34875	3	-	**glycoside hydrolase family 2**	48%	COG3250	G			**75**	**0**	**60**	**0**	**11**	**1**
		CCG34876	4	-	**sugar ABC transporter substratebinding protein**		COG1653	G			**69**	**6**	**66**	**3**	**6**	**1**
		CCG34877	5	-	**oligosaccharider ABC transporter permease**		COG0395	G			**72**	**0**	**61**	**0**	**3**	**0**
		CCG34878	6	-	**polysaccharide ABC transporter permease**		COG4209	G			**67**	**3**	**61**	**2**	**5**	**1**
		CCG34879	7	-	**glycoside hydrolase family 3**	59%	COG1472	G			**77**	**1**	**56**	**1**	**5**	**0**
		CCG34880	8	-	acylCoA thioesterase Ilike protein		None	None			63	0	55	2	3	0
		CCG34881	9	+	predicted protein		None	None			0	0	0	0	0	0
		CCG34882	10	+	hypothetical protein		None	None			47	1	39	3	5	0
		CCG34883	11	-	peptidylprolyl cistrans isomerase A		COG0652	O			66	0	61	0	1	0
		CCG34884	12	-	putative oligopeptide ABC transporter OppAIV		COG4166	E			70	9	69	17	5	1
		CCG34885	13	-	oligopeptide ABC transporter ATPbinding protein OppF		COG4608	E			36	15	50	9	2	1
		CCG34886	14	-	oligopeptide ABC transporter ATPbinding protein OppD		COG0444	EP			61	1	56	0	5	1
		CCG34887	15	-	putative oligopeptide ABC transporter permease protein OppC		COG1173	EP			69	0	66	1	2	0
		CCG34888	16	-	glutathione ABC transporter permease protein GsiC		COG0601	EP			62	0	60	0	4	0
		CCG34889	17	-	predicted protein		None	None			0	0	0	0	0	0
		CCG34890	18	+	Amidases related to nicotinamidase		COG1335	Q			45	0	41	0	4	0
		CCG34891	19	-	Uncharacterized MobArelated protein		COG2068	R			54	0	43	1	2	0
		CCG34892	20	-	Nterminal domain of molybdenumbinding protein		COG2005	R			30	10	25	0	0	0
		CCG34893	21	-	ABCtype cobalaminFe3siderophores transport systems ATPase components		COG1120	PH			57	0	43	8	3	0
		CCG34894	22	-	ABCtype Fe3siderophore transport system permease component		COG0609	P			54	0	45	1	5	0
		CCG34895	23	-	ABCtype Fe3hydroxamate transport system periplasmic component		COG0614	P			60	1	57	0	1	0
		CCG34896	24	+	Aerobictype carbon monoxide dehydrogenase middle subunit CoxMCutM homologs		COG1319	C			63	0	44	2	3	0
		CCG34897	25	+	Aerobictype carbon monoxide dehydrogenase small subunit CoxSCutS homologs		COG2080	C			39	0	26	0	2	0
		CCG34898	26	+	aldehyde oxidase and xanthine dehydrogenase molybdopterin binding domain protein		COG1529	C			69	0	57	1	6	0

**F4<br>XOS<br>*Bifidobacterium adolescentis***	**F4<br>31477 bp<br>HE717016**	CCG47748	1	-	50S ribosomal protein L5;		COG0094	J	YP_909196	Bifidobacterium adolescentis ATCC 15703	15	3	8	2	19	1
		CCG47749	2	-	50S ribosomal protein L24		COG0198	J	YP_909195	Bifidobacterium adolescentis ATCC 15703	9	4	5	0	1	0
		CCG47750	3	-	50S ribosomal protein L14		COG0093	J	YP_909194	Bifidobacterium adolescentis ATCC 15703	12	8	2	2	14	7
		CCG47751	4	-	30S ribosomal protein S17		COG0186	J	YP_909193	Bifidobacterium adolescentis ATCC 15703	0	0	0	0	0	0
		CCG47752	5	-	50S ribosomal protein L29		COG0255	J	YP_909192	Bifidobacterium adolescentis ATCC 15703	0	0	0	0	0	0
		CCG47753	6	-	50S ribosomal protein L16		COG0197	J	YP_909191	Bifidobacterium adolescentis ATCC 15703	13	0	6	0	9	0
		CCG47754	7	-	30S ribosomal protein S3		COG0092	J	YP_909190	Bifidobacterium adolescentis ATCC 15703	29	0	10	1	30	2
		CCG47755	8	-	50S ribosomal protein L22		COG0091	J	YP_909189	Bifidobacterium adolescentis ATCC 15703	14	11	5	5	19	1
		CCG47756	9	-	30S ribosomal protein S19		COG0185	J	YP_909188	Bifidobacterium adolescentis ATCC 15703	0	0	0	0	0	0
		CCG47757	10	-	50S ribosomal protein L2		COG0090	J	YP_909187	Bifidobacterium adolescentis ATCC 15703	27	3	9	3	24	0
		CCG47758	11	-	50S ribosomal protein L23		COG0089	J	YP_909186	Bifidobacterium adolescentis ATCC 15703	0	0	0	0	0	0
		CCG47759	12	-	50S ribosomal protein L4		COG0088	J	YP_909185	Bifidobacterium adolescentis ATCC 15703	27	3	9	0	18	0
		CCG47760	13	-	50S ribosomal protein L3		COG0087	J	YP_909184	Bifidobacterium adolescentis ATCC 15703	27	3	11	0	24	0
		CCG47761	14	-	30S ribosomal protein S11		COG0051	J	YP_909183	Bifidobacterium adolescentis ATCC 15703	0	0	0	0	0	0
		CCG47762	15	-	bifunctional acetaldehydeCoAalcohol dehydrogenase		COG1454	C	YP_909182	Bifidobacterium adolescentis ATCC 15703	47	2	18	2	33	0
		CCG47763	16	+	*ROK family transcriptional regulator*		COG1940	KG			12	0	4	2	5	1
		CCG47764	17	-	glyoxalase family protein		None	None		Bifidobacterium angulatum DSM 20098	2	2	2	1	2	0
		CCG47765	18	-	arabinose efflux permease		COG2814	G		Bifidobacterium breve DSM 20213	7	4	5	4	5	1
		CCG47766	19	-	zincbinding dehydrogenase		COG1063	ER	YP_909176	Bifidobacterium adolescentis ATCC 15703	6	5	2	1	2	0
		CCG47767	20	+	glycoside hydrolase family13	43%	COG1523	G		Bifidobacterium adolescentis L232	30	10	17	8	7	5
		CCG47768	21	-	predicted protein		None	None			0	0	0	0	0	0
		CCG47769	22	-	30S ribosomal protein S9		COG0103	J	YP_909173	Bifidobacterium adolescentis ATCC 15703	26	5	5	5	27	1
		CCG47770	23	-	50S ribosomal protein L13		COG0102	J	YP_909172	Bifidobacterium adolescentis ATCC 15703	15	1	10	1	1	0
		CCG34946	24	-	**glycoside hydrolase family 77**	34%	COG1640	G	YP_909171	Bifidobacterium adolescentis ATCC 15703	**39**	**1**	**16**	**2**	**7**	**1**
		CCG47771	25	-	***AbrB family trancriptional regulator***		None	None	YP_909170	Bifidobacterium adolescentis ATCC 15703	**21**	**3**	**14**	**1**	**3**	**1**
		CCG47772	26	-	**hypothetical protein BAD_0306**		COG1739	S	YP_909169	Bifidobacterium adolescentis ATCC 15703	**25**	**2**	**15**	**0**	**3**	**1**
		CCG47773	27	-	**2hydroxyacid dehydrogenase**		COG0111	HE	YP_909168	Bifidobacterium adolescentis ATCC 15703	**30**	**1**	**13**	**4**	**2**	**2**
		CCG47774	28	-	**bacterial capsule synthesis protein PGA_cap**		COG2843	M	YP_909167	Bifidobacterium adolescentis ATCC 15703	**29**	**0**	**11**	**2**	**4**	**0**
		CCG47775	29	-	**DNAdamageinducible protein P**		COG0389	L	YP_909166	Bifidobacterium adolescentis ATCC 15703	**34**	**4**	**17**	**5**	**7**	**0**
		CCG47776	30	-	**predicted protein**		None	None			**0**	**0**	**0**	**0**	**0**	**0**
		CCG47777	31	-	**putative esterase**		COG2272	I	YP_909165	Bifidobacterium adolescentis ATCC 15703	**41**	**0**	**17**	**1**	**22**	**0**
		CCG47778	32	-	**glycoside hydrolase family43**	97%	COG3507	G	YP_909164	Bifidobacterium adolescentis ATCC 15703	**44**	**0**	**21**	**3**	**22**	**0**
		CCG47779	33	-	**glycoside hydrolase family 8**	99%	COG3405	G	YP_909163	Bifidobacterium adolescentis ATCC 15703	**39**	**1**	**21**	**2**	**17**	**0**
		CCG47780	34	-	**glycoside hydrolase family 120**	99%	None	None	YP_909162	Bifidobacterium adolescentis ATCC 15703	**23**	**0**	**6**	**0**	**5**	**0**
**F5<br>XOS<br>*Bacteroides vulgatus***	**F5<br>39094 bp<br>HE717017**	CCG34969	1	-	**glycoside hydrolase family43**	53%	None	G	YP_001297384	Bacteroides vulgatus ATCC 8482	32	23	75	48	6	3
		CCG34970	2	+	*twocomponent system sensor histidine kinaseresponse regulator*		COG0642	T	YP_001297385	Bacteroides vulgatus ATCC 8482	113	17	124	60	19	12
		CCG34971	3	+	transposase IS4 family		None	None	ZP_05258040	Bacteroides sp. 4_3_47FAA	0	0	0	0	0	0
		CCG34972	4	+	transposase IS4 family		None	None	ZP_05258037	Bacteroides sp. 4_3_47FAA	17	21	25	17	4	1
		CCG34973	5	-	transposase		COG5433	L	ZP_05257946	Bacteroides sp. 4_3_47FAA	35	1	33	0	10	0
		CCG34974	6	-	transposase		None	None	YP_001297506	Bacteroides vulgatus ATCC8482	0	0	0	0	0	0
		CCG34975	7	-	**RagBSusD domaincontaining protein**		None	None	ZP_05254855	Bacteroides sp. 4_3_47FAA	**98**	**10**	**126**	**32**	**12**	**4**
		CCG34976	8	-	**TonBdependent receptor**		COG1629	P	ZP_05254856	Bacteroides sp. 4_3_47FAA	**98**	**16**	**127**	**71**	**19**	**15**
		CCG34977	9	-	**glycoside hydrolase family43**	46%	COG3507	G	YP_001297391	Bacteroides vulgatus ATCC8482	**85**	**29**	**126**	**78**	**10**	**7**
		CCG34978	10	-	**glycoside hydrolase family43**	81%	COG3508	G	YP_001297392	Bacteroides vulgatus ATCC 8482	**71**	**27**	**118**	**41**	**8**	**1**
		CCG34979	11	-	**glycoside hydrolase family10**	65%	COG3693	G	YP_001297393	Bacteroides vulgatus ATCC 8482	**99**	**5**	**124**	**8**	**9**	**2**
		CCG34980	12	+	transposase		COG3547	L	YP_001297394	Bacteroides vulgatus ATCC 8482	48	2	65	1	4	0
		CCG34981	13	-	sugar GlycosidePentosideHexuronide transporter		COG2211	G	YP_001297395	Bacteroides vulgatus ATCC 8482	94	14	125	26	13	2
		CCG34982	14	-	IS3 element transposase		None	None		Shigella flexneri 2a str. 301	0	0	0	0	0	0
		CCG34983	15	-	glycoside hydrolase family16	34%	COG2273	G	YP_001297396	Bacteroides vulgatus ATCC8482	91	4	108	7	4	1
		CCG34984	16	-	phosphoglucomutasephosphomannomutase		COG1109	G	YP_001297398	Bacteroides vulgatus ATCC 8482	84	21	122	23	13	7
		CCG34985	17	-	putative lipoprotein		None	None	YP_001297399	Bacteroides vulgatus ATCC 8482	82	4	90	2	6	0
		CCG34986	18	-	putative exopolyphosphataserelated protein		COG0618	R	YP_001297400	Bacteroides vulgatus ATCC 8482	86	11	123	27	7	1
		CCG34987	19	-	ribulosephosphate 3epimerase		COG0036	G	YP_001297402	Bacteroides vulgatus ATCC 8482	73	10	91	18	8	1
		CCG34988	20	-	RNA polymerase ECFtype sigma factor		None	None	YP_001297403	Bacteroides vulgatus ATCC 8482	109	2	124	8	10	2
		CCG34989	21	-	RNA polymerase ECFtype sigma factor		COG1595	K	YP_001297404	Bacteroides vulgatus ATCC 8482	71	45	113	69	2	0
		CCG34990	22	+	putative lipoprotein		None	None	YP_001297405	Bacteroides vulgatus ATCC 8482	75	20	125	38	9	3
		CCG34991	23	-	methionyltRNA formyltransferase		COG0223	J	YP_001297406	Bacteroides vulgatus ATCC 8482	72	28	121	63	12	6
		CCG34992	24	-	putative chloride channel protein		COG0038	P	YP_001297407	Bacteroides vulgatus ATCC 8482	89	34	131	109	14	4
		CCG34993	25	-	*putative translation factor*		COG0009	J	YP_001297408	Bacteroides vulgatus ATCC 8482	57	34	107	69	11	5
		CCG34994	26	-	carbohydrate esterase family9		COG1820	G	YP_001297409	Bacteroides vulgatus ATCC 8482	87	10	125	30	9	3
		CCG34995	27	-	drug efflux protein		COG0841	V	YP_001297410	Bacteroides vulgatus ATCC 8482	96	21	126	71	15	5
**F6<br>GOS<br>*Bifidobacterium longum***	**F6<br>37418 bp<br>HE717012**	CCG34844	1	+	Carbohydrate Binding module family 48-glycoside hydrolase family13	no hit	COG1523	G	NP_696154	Bifidobacterium longum NCC2705	24	4	10	2	43	8
		CCG34845	2	+	hypothetical protein Blon03001741		COG2461	S	NP_696153	Bifidobacterium longum NCC2705	0	0	0	0	0	0
		CCG34846	3	+	*putative transcriptional regulator*		COG1396	K	NP_696153	Bifidobacterium longum NCC2705	15	0	4	0	31	2
		CCG34847	4	+	conserved hypothetical protein		COG4832	S	NP_696152	Bifidobacterium longum NCC2705	13	2	9	6	30	9
		CCG34848	5	-	predicted protein		COG3180	R		Bifidobacterium longum subsp. infantis CCUG 52486	0	0	0	0	0	0
		CCG34849	6	-	DNA3methyladenine glycosylase I		COG2818	L	NP_696151	Bifidobacterium longum NCC2705	14	6	4	2	26	4
		CCG34850	7	-	**glycoside hydrolase family2**	100%	COG3250	G	NP_696150	Bifidobacterium longum NCC2705	**21**	**10**	**8**	**5**	**37**	**10**
		CCG34851	8	+	hypothetical protein BL0977		COG4936	TK	NP_696149	Bifidobacterium longum NCC2705	0	0	0	0	0	0
		CCG34852	9	+	galactoside symporter		COG2211	G	NP_696148	Bifidobacterium longum NCC2705	15	6	9	3	46	9
		CCG34853	10	+	**glycoside hydrolase family42**	95%	COG1874	G		Bifidobacterium longum DJO10A	6	3	2	1	36	10
		CCG34854	11	+	hypothetical protein BLD_0725		COG4065	S		Bifidobacterium longum DJO10A	0	0	0	0	0	0
		CCG34855	12	+	Uncharacterized conserved protein		COG3371	S	NP_696145	Bifidobacterium longum NCC2705	18	1	6	3	35	19
		CCG34856	13	-	5enolpyruvylshikimate3phosphate synthase		COG0128	E	NP_696144	Bifidobacterium longum NCC2705	21	1	7	2	42	15
		CCG34857	14	-	*acetate kinase*		COG0282	C	NP_696143	Bifidobacterium longum NCC2705	20	2	8	3	47	13
		CCG34858	15	-	phosphate acetyltransferase		COG0280	C	NP_696142	Bifidobacterium longum NCC2705	19	2	8	1	39	12
		CCG34859	16	-	Fructose26bisphosphatase		COG0406	G	NP_696141	Bifidobacterium longum NCC2705	13	0	7	5	38	5
		CCG34860	17	-	bifunctional nicotinatenucleotide adenylyltransferase NadDhypothetical protein		COG0799	S	NP_696140	Bifidobacterium longum NCC2705	6	3	1	1	21	2
		CCG34861	18	-	unctional Nacetylglucosamine1phosphate uridyltransferaseglucosamine1phosphate acetyltransferase		COG1207	M	NP_696139	Bifidobacterium longum NCC2705	16	0	5	2	43	3
		CCG34862	19	-	*ribosephosphate pyrophosphokinase*		COG0462	FE	NP_696138	Bifidobacterium longum NCC2705	17	5	2	1	47	15
		CCG34863	20	-	acyltransferase		COG1835	I	NP_696137	Bifidobacterium longum NCC2705	29	0	8	1	39	10
		CCG34864	21	-	ATPdependent Zn protease		COG4868	S		Bifidobacterium longum subsp. infantis CCUG 52486	16	6	5	2	23	11
		CCG34865	22	+	GMP synthase		COG0519	F	NP_696136	Bifidobacterium longum NCC2705	25	9	5	5	49	13
		CCG34866	23	-	xylulose5phosphatefructose6phosphate phosphoketolase		COG3957	G	NP_696135	Bifidobacterium longum NCC2705	32	2	10	7	43	15
		CCG34867	24	+	possible HAD hydrolase		COG0561	R	NP_696134	Bifidobacterium longum NCC2705	14	1	6	0	35	7
		CCG34868	25	+	ABCtype transport system protein		COG1464	P	NP_696133	Bifidobacterium longum NCC2705	16	7	6	0	44	10
		CCG34869	26	+	ATP binding protein of ABC transporter		COG1135	P	NP_696132	Bifidobacterium longum NCC2705	13	4	7	7	45	13
		CCG34870	27	+	ABCtype metal ion transport system permease component		COG2011	P	NP_696131	Bifidobacterium longum NCC2705	15	0	2	1	45	7
		CCG34871	28	+	Metaldependent amidaseaminoacylasecarboxypeptidase		COG1473	R	NP_696130	Bifidobacterium longum NCC2705	12	7	4	3	46	13
		CCG34872	29		NAD synthase		COG0171	H	NP_696129	Bifidobacterium longum NCC2705	13	6	5	7	42	12

					ILEUM MUCOSA											
I7 XOS Bacteroidales*	I7 37821 bp HE717006	CCG34736	1	-	Glycosyl hydrolase family 115	36%	COG3669	G			**13**	**0**	**2**	**0**	**1**	**0**
		CCG34737	2	+	lipolytic enzyme GDSL		COG2755	E			12	0	2	0	1	1
		CCG34738	3	+	**GPH family xyloside:cation symporter**		COG2211	G			**13**	**0**	**2**	**0**	**1**	**0**
		CCG34739	4	+	**Glycosyl hydrolase family 10**	60%	COG3693	G			**13**	**0**	**2**	**0**	**1**	**0**
		CCG34740	5	+	**Glycosyl hydrolase family 43**	77%	COG3507	G			**11**	**0**	**2**	**0**	**2**	**0**
		CCG34741	6	-	RNA polymerase ECFtype sigma factor		COG1595	K			12	2	2	0	0	0
		CCG34742	7	-	pectin lyase foldvirulence factor		COG3893	L			14	0	2	0	1	0
		CCG34743	8	-	conserved hypothetical protein		COG2966	S			10	0	2	0	0	0
		CCG34744	9	-	putative outer membrane protein probably involved in nutrient binding		COG1643	L			13	0	2	0	2	0
		CCG34745	10	-	putative outer membrane protein probably involved in nutrient binding		COG4206	H			13	0	2	0	2	1
		CCG34746	11	-	putative lipoprotein		COG0243	C			14	0	2	0	0	0
		CCG34747	12	-	hypothetical protein PBR_1732		COG0109	O			14	0	2	0	0	0
		CCG34748	13	-	RHS repeatassociated core domain protein		COG4993	G			9	0	2	0	0	0
		CCG34749	14	+	predicted protein		None	None			0	0	0	0	0	0
		CCG34750	15	-	RHS repeatassociated core domain protein		COG3209	M			7	0	2	0	0	0
		CCG34751	16	-	RHS repeatassociated core domain protein		COG3209	M			1	1	0	0	0	0
		CCG34752	17	-	*twocomponent system sensor histidine kinaseresponse regulator*		COG0642	T			14	0	2	0	0	0
		CCG34753	18	-	TonBdependent outer membrane receptor		COG4771	P			16	0	2	0	0	0
I8 XOS Bacteroidales*	I8 38518 bp HE717007	CCG34754	1	-	SusC outer membrane protein involved in starch binding		COG4206	H			13	0	2	0	2	0
		CCG34755	2	-	glycoside hydrolase family 35	31%	COG1874	G			13	0	2	0	2	0
		CCG34756	3	-	glycoside hydrolase family 67	57%	COG3661	G			13	0	2	0	2	0
		CCG34757	4	-	*sensor histidine kinaseresponse regulator*		COG0642	T			14	0	2	0	2	0
		CCG34758	5	-	Glycosyde hydrolase family 115	36%	COG3669	G			13	0	2	0	1	0
		CCG34759	6	+	lipolytic enzyme GDSL		COG2755	E			12	0	2	0	1	1
		CCG34760	7	+	**GPH family xyloside:cation symporter**		COG2211	G			**13**	**0**	**2**	**0**	**1**	**0**
		CCG34761	8	+	**Glycosyde hydrolase family 10**	60%	COG3693	G			**13**	**0**	**2**	**0**	**1**	**0**
		CCG34762	9	+	**glycoside hydrolase family43**	77%	COG3507	G			**12**	**0**	**2**	**0**	**2**	**0**
		CCG34763	10	-	RNA polymerase ECFtype sigma factor		COG1595	K			12	2	2	0	0	0
		CCG34764	11	-	hypothetical protein		COG3893	L			14	0	2	0	1	0
		CCG34765	12	-	conserved hypothetical protein		COG2966	S			10	0	2	0	0	0
		CCG34766	13	-	putative outer membrane protein probably involved in nutrient binding		COG1643	L			13	0	2	0	2	0
		CCG34767	14	-	putative outer membrane protein probably involved in nutrient binding		COG4206	H			13	0	2	0	2	0
		CCG34768	15	-	putative lipoprotein		COG0243	J			14	0	2	0	0	0
		CCG34769	16	-	hypothetical protein PBR_1732		COG0109	O			14	0	2	0	0	0
		CCG34770	17	-	RHS repeatassociated core domain protein		COG4993	G			9	0	2	0	0	0
		CCG34771	18	+	predicted protein		None	None			0	0	0	0	0	0
		CCG34772	19	-	RHS repeatassociated core domain protein		COG3846	U			0	0	0	0	0	0
I9 FOS Bacteria*	I9a 14714 bp HE717008	CCG34773	1	+	predicted protein		COG2804	NU			0	0	0	0	0	0
		CCG34774	2	+	*Pyridoxine kinase*		COG2240	H	ZP_01996253	Dorea longicatena DSM 13814	18	1	12	0	0	1
		CCG34775	3	-	**Glycoside Hydrolase Family 32**	38%	COG1621	G	ZP_01996254	Dorea longicatena DSM 13814	**19**	**0**	**14**	**1**	**0**	**4**
		CCG34776	4	-	**PTS system glucose subfamily IIA component**		COG1263	G	ZP_01996255	Dorea longicatena DSM 13814	**25**	**0**	**12**	**1**	**4**	**0**
		CCG34777	5	-	***transcriptional regulator LacI family***		COG1609	K	ZP_01996256	Dorea longicatena DSM 13814	**12**	**3**	**9**	**4**	**1**	**1**
		CCG34778	6	-	**ABC transporter**		COG1132	V	ZP_01996257	Dorea longicatena DSM 13814	**32**	**1**	**15**	**3**	**4**	**0**
		CCG34779	7	-	**ABCtype multidrug transport system ATPase and permease components**		COG1132	V	ZP_01996258	Dorea longicatena DSM 13814	**28**	**0**	**16**	**4**	**5**	**0**
		CCG34780	8	+	*transcriptional regulator LysR family*		COG0583	K	ZP_01996259	Dorea longicatena DSM 13814	7	0	7	0	2	0
		CCG34781	9	-	hypothetical protein DORLON_02268		COG5074	U	ZP_01996261	Dorea longicatena DSM 13814	10	5	10	1	1	0
		CCG34782	10	-	*transcriptional regulator XRE family*		COG3655	K	ZP_01996262	Dorea longicatena DSM 13814	0	0	0	0	0	0
		CCG34783	11	-	xanthine permease		COG2233	F	ZP_01996263	Dorea longicatena DSM 13814	19	1	9	0	6	1
		CCG34784	12	-	Putative transposase YhgAlike		COG1129	G	ZP_01996264	Dorea longicatena DSM 13814	12	1	8	1	4	2
	I9b 13700 bp HE717009	CCG34785	1	-	DNA methylase		COG0827	L	ZP_03288401	Clostridium nexile DSM 1787	116	0	117	0	21	0
		CCG34786	2	-	50S ribosomal protein L23		COG1488	H	ZP_03288400	Clostridium nexile DSM 1788	70	0	61	0	1	0
		CCG34787	3	-	hypothetical protein CLONEX_00589		None	None	ZP_03288399	Clostridium nexile DSM 1787	0	0	0	0	0	0
		CCG34788	4	-	conserved hypothetical protein		COG5498	M	ZP_03288398	Clostridium nexile DSM 1788	70	0	93	0	8	0
		CCG34789	5	-	Cell wall associated hydrolases invasionassociated proteins		COG0791	M	ZP_03288397	Clostridium nexile DSM 1787	90	0	103	1	16	0
		CCG34790	6	-	Acetyltransferase GNAT family		COG0526	OC			9	7	7	5	0	0
		CCG34791	7	-	Domain of unknown function DUF87		COG3451	U	ZP_03288396	Clostridium nexile DSM 1787	56	0	0	0	16	0
		CCG34792	8	-	hypothetical protein BACPEC_00198		COG2959	H	ZP_03288395	Clostridium nexile DSM 1788	35	1	32	0	4	0
		CCG34793	9	-	hypothetical protein CLONEX_00584		COG5598	H	ZP_03288394	Clostridium nexile DSM 1787	32	1	53	0	0	0
		CCG34794	10	-	hypothetical protein BACPEC_00196		COG5346	S	ZP_03288393	Clostridium nexile DSM 1788	38	1	56	1	6	0
		CCG34795	11	-	putative electron transport complex RnfABCDGE type A subunit		COG1933	L		Eubacterium rectale DSM 17629	6	0	3	1	0	0

**I10 FOS *Eubacterium rectale***	**I10a 11660 bp HE717015**	CCG34929	1	-	ABC transporter permease protein		None	None	YP_002936193	Eubacterium rectale ATCC 33656	0	0	0	0	0	0
		CCG34930	2	+	**ABC transporter permease protein**		COG0395	G	YP_002936193	Eubacterium rectale ATCC 33656	**65**	**1**	**82**	**1**	**3**	**0**
		CCG34931	3	+	**ABC transporter substrate binding protein**		COG1653	G	YP_002936194	Eubacterium rectale ATCC 33656	**92**	**0**	**108**	**11**	**9**	**1**
		CCG34932	4	+	**glycoside hydrolase family 32**	37%	COG1621	G	YP_002936195	Eubacterium rectale ATCC 33656	**88**	**0**	**107**	**0**	**7**	**0**
		CCG34933	5	+	***pfkBtype carbohydrate kinase family protein***		COG0524	G	YP_002936196	Eubacterium rectale ATCC 33656	**90**	**2**	**98**	**13**	**3**	**1**
		CCG34934	6	+	ATPaselike protein		COG1209	M	YP_002936197	Eubacterium rectale ATCC 33656	49	38	105	97	3	1
		CCG34935	7	+	Sulfate permease and related transporters MFS superfamily		COG0659	P	YP_002936198	Eubacterium rectale ATCC 33656	83	2	94	17	7	0
		CCG34936	8	+	Sulfate permease and related transporters MFS superfamily		COG3407	I	YP_002936198	Eubacterium rectale ATCC 33656	78	0	82	0	3	0
		CCG34937	9	+	ATPdependent DNA helicase RecQ family		COG0514	L	YP_002936199	Eubacterium rectale ATCC 33656	95	0	108	0	12	0
		CCG34938	10	+	Predicted membrane protein		COG4720	S	YP_002936200	Eubacterium rectale ATCC 33656	56	25	83	21	5	0
	**I10b 12461 bp HE717011**	CCG34829	1	-	Phosphoribosylanthranilate isomerase		COG0135	E	YP_002936201	Eubacterium rectale ATCC 33656	55	2	81	4	3	0
		CCG34830	2	+	hypothetical protein		COG1363	G	YP_002936202	Eubacterium rectale ATCC 33656	79	0	90	1	3	0
		CCG34831	3	+	Lactoylglutathione lyase and related lyases		COG0346	E	YP_002936203	Eubacterium rectale ATCC 33656	21	0	30	0	1	0
		CCG34832	4	+	Predicted DNAbinding protein with PD1like DNAbinding motif		COG1661	R	YP_002936212	Eubacterium rectale ATCC 33656	18	1	46	4	1	0
		CCG34833	5	+	DNAdamageinducible protein		COG3071	H	YP_002936213	Eubacterium rectale ATCC 33656	28	1	30	0	0	0
		CCG34834	6	+	glycosyl transferase family 2		COG0463	M			12	1	14	4	0	0
		CCG34835	7	+	hypothetical protein EUBREC_0276		COG3738	S	YP_002936214	Eubacterium rectale ATCC 33656	68	0	92	0	3	0
		CCG34836	8	-	hypothetical protein		COG5543	S	CBK95362	Eubacterium rectale M1041	0	0	0	0	0	0
		CCG34837	9	-	Transposase and inactivated derivatives		COG3464	L	CBK95361	Eubacterium rectale M1041	28	24	56	41	8	4
		CCG34838	10	+	putative lipoprotein		COG4880	R		Eubacterium hallii DSM 3353	84	4	73	7	1	0
		CCG34839	11	+	GTPbinding signal recognition particle SRP54 Gdomain		COG2098	S	ZP_02041825	Ruminococcus gnavus ATCC 29149	0	0	0	0	0	0
		CCG34840	12	-	ABCtype antimicrobial peptide transport system ATPase component		COG1136	V	CBK92298	Eubacterium rectale M1041	0	0	0	0	0	0
		CCG34841	13	-	ABCtype antimicrobial peptide transport system ATPase component		COG1136	V	CBK92299	Eubacterium rectale M1042	101	1	115	2	2	0
		CCG34842	14	-	hypothetical protein CLOL250_01817		COG4652	S	ZP_02075041	Clostridium sp. L250	144	98	133	133	10	2
		CCG34843	15	-	hypothetical protein RUMGNA_02594		COG1110	L	ZP_02075040	Clostridium sp. L251	0	0	0	0	0	0
**I11 Lactulose GOS *Streptococcus thermophilus***	**I11 30347 bp HE717018**	CCG34996	1	-	glycosyltransferase family 2 and 8		COG0463	M	YP_820716	Streptococcus thermophilus LMD9	4	0	0	0	2	0
		CCG34997	2	+	ABCtype antimicrobial peptide transport system permease component		COG4591	M	YP_820717	Streptococcus thermophilus LMD9	1	0	0	0	1	0
		CCG34998	3	+	ABCtype antimicrobial peptide transport system ATPase component		COG1136	V	YP_820718	Streptococcus thermophilus LMD9	1	0	0	0	1	0
		CCG34999	4	+	hypothetical protein STER_1350		COG0540	F	YP_820720	Streptococcus thermophilus LMD9	0	0	0	0	0	0
		CCG35000	5	+	hypothetical protein STER_1351		COG3941	R	YP_820721	Streptococcus thermophilus LMD9	0	0	0	0	0	0
		CCG35001	6	-	Nadriven multidrug efflux pump		COG0534	V	YP_820722	Streptococcus thermophilus LMD9	1	1	0	0	1	0
		CCG35002	7	-	MoaANirJ family FeS oxidoreductase		COG0535	R		Streptococcus thermophilus ND03	0	0	0	0	0	0
		CCG35003	8	-	transporter		COG2223	P	YP_820724	Streptococcus thermophilus LMD9	1	1	0	0	0	0
		CCG35004	9	-	radical SAM superfamily protein		COG0535	R	YP_820725	Streptococcus thermophilus LMD9	1	1	0	0	3	1
		CCG35005	10	-	*XRE family transcriptional regulator*		COG1395	K	YP_820727	Streptococcus thermophilus LMD9	0	0	0	0	1	0
		CCG35006	11	-	transposase		COG2801	L	YP_820728	Streptococcus thermophilus LMD9	1	1	0	0	0	0
		CCG35007	12	-	transposase		COG2963	L	YP_820729	Streptococcus thermophilus LMD9	0	0	0	0	0	0
		CCG35008	13	-	hypothetical protein STER_1361;		COG5453	S	YP_820730	Streptococcus thermophilus LMD9	0	0	0	0	0	0
		CCG35009	14	-	Parvulinlike peptidylprolyl isomerase		COG2054	R	YP_820732	Streptococcus thermophilus LMD9	0	0	0	0	2	0
		CCG35010	15	-	ATPase involved in DNA repair		COG0419	L	YP_820733	Streptococcus thermophilus LMD9	5	0	0	0	9	1
		CCG35011	16	-	ATPase involved in DNA repair		COG0419	L	YP_820733	Streptococcus thermophilus LMD9	5	0	0	0	4	1
		CCG35012	17	-	ATPdependent dsDNA exonuclease		COG0420	L	YP_820734	Streptococcus thermophilus LMD9	4	1	1	1	5	0
		CCG35013	18	-	**glycoside hydrolase family 2**	100%	COG3250	G	YP_820735	Streptococcus thermophilus LMD9	**11**	**0**	**1**	**1**	**35**	**4**
		CCG35014	19	-	**Lactose transport protein**		COG2211	G	YP_820736	Streptococcus thermophilus LMD9	**7**	**1**	**1**	**0**	**14**	**3**
		CCG35015	20	-	**Aldose 1epimerase**		COG2017	G		Streptococcus thermophilus	**6**	**0**	**0**	**0**	**4**	**0**
		CCG35016	21	-	**UDPglucose 4epimerase**		COG1087	M	YP_820738	Streptococcus thermophilus LMD9	**3**	**0**	**0**	**0**	**5**	**0**
		CCG35017	22	-	**Galactose1phosphate uridyltransferase**		COG4468	G	YP_820739	Streptococcus thermophilus LMD9	**8**	**1**	**0**	**0**	**14**	**0**
		CCG35018	23	-	***galactokinase***		COG0153	G	YP_820740	Streptococcus thermophilus LMD9	**5**	**1**	**0**	**0**	**10**	**0**
		CCG35019	24	+	***galactose operon repressor***		COG1609	K	YP_820741	Streptococcus thermophilus LMD9	**1**	**0**	**0**	**0**	**1**	**1**
**I12 Lactulose Clostridiales***	**I12 13317 bp HE717019**	CCG35020	1	-	phosphoserine phosphatasehomoserine phosphotransferase bifunctional protein		COG0560	E		Ruminococcus torques L214	2	2	1	1	0	0
		CCG35021	2	+	predicted protein		COG3829	KT			0	0	0	0	0	0
		CCG35022	3	-	**Glycoside hydrolase family 42**	49%	COG1874	G			**14**	**0**	**6**	**1**	**1**	**0**
		CCG35023	4	-	**Glycoside hydrolase family 2**	46%	COG3250	G			**62**	**0**	**30**	**5**	**13**	**1**
		CCG35024	5	-	**lactose transport system permease protein LacG**		COG0395	G			**40**	**0**	**17**	**0**	**2**	**0**
		CCG35025	6	-	**lactose transport system permease protein LacF**		COG1175	G			**39**	**4**	**22**	**8**	**3**	**0**
		CCG35026	7	-	**lactose ABC superfamily ATP binding cassette transporter binding protein**		COG1653	G			**44**	**13**	**30**	**23**	**7**	**1**
		CCG35027	8	-	**AraCtype DNAbinding domaincontaining proteins**		COG2207	KT			**24**	**4**	**17**	**7**	**4**	**0**
		CCG35028	9	-	**putative mannose6phosphate isomerase**		COG0662	G			**0**	**0**	**0**	**0**	**0**	**0**
		CCG35029	10	-	bacitracin transport ATPbinding protein BcrA family protein		COG1131	V			31	0	12	1	3	0
		CCG35030	11	-	predicted protein		COG4713	S			34	3	21	9	5	0
		CCG35031	12	-	predicted protein		COG4380	S			49	5	27	6	3	0

**I13 Lactulose GOS** ***Faecalibacterium prausnitzii***	**I13 32036 bp HE717014**	CCG34899	1	+	conserved hypothetical protein		COG0048	J	ZP_07800047	Faecalibacterium cf. prausnitzii KLE1255	2	0	1	0	0	0
		CCG34900	2	+	**Glycoside hydrolase family2**	no hit	COG3250	G	ZP_07800045	Faecalibacterium cf. prausnitzii KLE1255	**1**	**0**	**5**	**0**	**16**	**1**
		CCG34901	3	+	Site specific recombinases DNA invertase Pin homologs		COG1961	L	ZP_07800044	Faecalibacterium cf. prausnitzii KLE1256	1	0	3	0	4	0
		CCG34902	4	+	putative transposase insK for insertion sequence element IS152		COG2801	L			2	1	7	0	7	3
		CCG34903	5	+	Sitespecific recombinases DNA invertase Pin homologs		COG1196	D	ZP_07800044	Faecalibacterium cf. prausnitzii KLE1255	9	0	5	0	4	0
		CCG34904	6	-	transposase IS200family protein		COG5537	D		Faecalibacterium prausnitzii M212	3	0	2	0	0	0
		CCG34905	7	-	purine nucleoside phosphorylase I inosine and guanosinespecific		COG0005	F		Faecalibacterium prausnitzii L26	4	0	9	0	1	0
		CCG34906	8	-	Glycoside hydrolase family77	no hit	COG1640	G			4	0	6	0	8	0
		CCG34907	9	-	Metaldependent hydrolase		COG3568	R			32	0	22	0	2	0
		CCG34908	10	-	phosphotransferase system EIIC component		COG1263	G			1	0	4	0	12	0
		CCG34909	11	+	*transcriptional regulator LacI family*		COG1609	K			17	0	21	0	4	0
		CCG34910	12	-	*transcriptional regulator AraC family*		COG2207	K	ZP_07798578	Faecalibacterium cf. prausnitzii KLE1255	0	0	0	0	7	0
		CCG34911	13	+	Glycosyltransferase Family 35		COG0058	G	ZP_07798576	Faecalibacterium cf. prausnitzii KLE1255	28	0	20	0	22	0
		CCG34912	14	-	putative precorrin8X methylmutase		COG2082	H	ZP_07800000	Faecalibacterium cf. prausnitzii KLE1255	22	0	13	0	1	0
		CCG34913	15	-	cobyric acid synthase CobQ		COG1492	H	ZP_07800001	Faecalibacterium cf. prausnitzii KLE1255	3	0	5	0	3	0
		CCG34914	16	-	Histidinolphosphatearomatic aminotransferase and cobyric acid decarboxylase		COG0079	E			20	0	20	0	2	0
		CCG34915	17	-	cobalamin biosynthesis protein CobD		COG1270	H	ZP_07800003	Faecalibacterium cf. prausnitzii KLE1255	2	0	2	0	10	0
		CCG34916	18	-	Fructose26bisphosphatase		COG0406	G	ZP_07800004	Faecalibacterium cf. prausnitzii KLE1255	48	0	53	0	12	0
		CCG34917	19	-	adenosylcobalamin biosynthesis protein CobU		COG2087	H			21	0	19	0	0	0
		CCG34918	20	-	cobalamin5phosphate synthase		COG0368	H	ZP_07800006	Faecalibacterium cf. prausnitzii KLE1255	24	0	28	0	0	0
		CCG34919	21	-	putative adenosylcobinamide kinaseadenosylcobinamidephosphate guanylyltransferase		COG2087	H	ZP_07800007	Faecalibacterium cf. prausnitzii KLE1255	57	0	39	0	0	0
		CCG34920	22	-	nicotinatenucleotidedimethylbenzimidazole phosphoribosyltransferase		COG2038	H	ZP_07800008	Faecalibacterium cf. prausnitzii KLE1255	6	0	7	1	5	0
		CCG34921	23	-	cobyrinic Acid acdiamide synthase		COG1797	H			34	0	24	0	3	0
		CCG34922	24	-	precorrin6Y C515methyltransferase CbiT subunit		COG2242	H			1	0	14	0	0	0
		CCG34923	25	-	precorrin6x reductase		COG2099	H			0	0	0	0	1	0
		CCG34924	26	-	precorrin3B C17methyltransferase		COG1010	H			18	0	6	0	2	0
		CCG34925	27	-	protein CbiG		COG2073	H			3	2	5	3	2	0
		CCG34926	28	-	precorrin4 C11methyltransferase		COG2875	H	ZP_07800014	Faecalibacterium cf. prausnitzii KLE1255	11	2	18	0	1	0
		CCG34927	29	-	precorrin2 C20methyltransferase		COG2243	H			144	7	80	23	0	0
		CCG34928	30	-	cobalamin biosynthesis protein CbiD		COG1903	H	ZP_07800016	Faecalibacterium cf. prausnitzii KLE1255	0	0	0	0	0	0
**I14 Lactulose GOS** ***Streptococcus thermophilus***	**I14 26887 bp HE717020**	CCG35032	1	+	DNA translocase ftsk		COG1674	D		Streptococcus thermophilus ND03	4	0	0	0	4	1
		CCG35033	2	+	Carbohydrate Binding modules family 41 and 48	no hit	COG0296	G	ZP_07724042	Streptococcus vestibularis F0396	2	0	0	0	1	0
		CCG35034	3	+	Glycoside hydrolase family 13	no hit	COG1523	G	ZP_07724042	Streptococcus vestibularis F0396	0	0	0	0	0	0
		CCG35035	4	+	Glycoside hydrolase family 13	no hit	COG1523	G	ZP_07724042	Streptococcus vestibularis F0396	1	0	0	0	0	0
		CCG35036	5	-	Glutamatecysteine ligase putativeamino acid ligase putative		COG2918	H	YP_139841	Streptococcus thermophilus LMG 18311	3	0	1	1	9	0
		CCG35037	6	+	ABC transporter ATPbinding protein		COG1132	V	YP_139839	Streptococcus thermophilus LMG 18311	1	0	1	1	1	1
		CCG35038	7	-	ABC transporter ATP binding protein truncated		COG0488	R	YP_139837	Streptococcus thermophilus LMG 18311	0	0	0	0	0	0
		CCG35039	8	+	LPXTGmotif cell wall anchor domain protein		COG0098	J	YP_139835	Streptococcus thermophilus LMG 18311	0	0	0	0	0	0
		CCG35040	9	+	LPXTGmotif cell wall anchor domain protein		COG2326	S	YP_139834	Streptococcus thermophilus LMG 18311	5	0	1	1	2	0
		CCG35041	10	+	IS3Spn1 transposase		COG3424	Q	YP_139833	Streptococcus thermophilus LMG 18311	0	0	0	0	0	0
		CCG35042	11	-	***galactose operon repressor***		COG1609	K	YP_139832	Streptococcus thermophilus LMG 18311	**2**	**0**	**0**	**0**	**1**	**0**
		CCG35043	12	+	***galactokinase***		COG0153	G	YP_139831	Streptococcus thermophilus LMG 18311	**6**	**1**	**0**	**0**	**10**	**0**
		CCG35044	13	+	**galactose1phosphate uridylyltransferase**		COG4468	G	YP_139830	Streptococcus thermophilus LMG 18311	**8**	**0**	**0**	**0**	**15**	**0**
		CCG35045	14	+	**UDPglucose 4epimerase**		COG1087	M	YP_139829	Streptococcus thermophilus LMG 18311	**3**	**0**	**0**	**0**	**6**	**0**
		CCG35046	15	+	**Aldose 1epimerase**		COG2017	G	YP_139828	Streptococcus thermophilus LMG 18311	**5**	**0**	**0**	**0**	**4**	**0**
		CCG35047	16	+	**Lactose transport protein**		COG2211	G	YP_139827	Streptococcus thermophilus LMG 18311	**7**	**0**	**1**	**1**	**16**	**2**
		CCG35048	17	+	**glycoside hydrolase family 2**	100%	COG3250	G	YP_139826	Streptococcus thermophilus LMG 18311	**10**	**3**	**1**	**1**	**35**	**2**
		CCG35049	18	+	**transposase**		COG2963	L	YP_820729	Streptococcus thermophilus LMD9	**0**	**0**	**0**	**0**	**0**	**0**
		CCG35050	19	+	**transposase**		COG2801	L	YP_820728	Streptococcus thermophilus LMD9	**1**	**1**	**0**	**0**	**0**	**0**
		CCG35051	20	+	***XRE family transcriptional regulator***		COG1395	K	YP_820727	Streptococcus thermophilus LMD9	**0**	**0**	**0**	**0**	**1**	**0**
		CCG35052	21	+	**radical SAM superfamily protein**		COG0535	R	YP_820725	Streptococcus thermophilus LMD9	**1**	**0**	**0**	**0**	**2**	**0**
		CCG35053	22	-	predicted protein		None	None			0	0	0	0	0	0

Column A: Clone number and taxonomic assignment by BLASTX analysis against the non redundant NCBI database (E-value ≤ 10-8, identity ≥ 90%, query length coverage ≥50%).
Contigs were assigned to a class, genus or species, only if at least 50 % of the ORFs were assigned to the same organism. * Megan based taxonomic assignment

Column B: Contig number and size

Column C: ORF accession number

Column D: ORF position on the contig

Column E: DNA strand

Column F: ORFs were inferred and manually annotated, based on BLASTX analysis against the NCBI non redundant and environmental database (E-value < 10-8, identity > 35%, query length coverage ≥50%), and BLASTP analysis against the CAZy data base.
In bold, proteins assigned to prebiotic metabolization pathways; in italics : putative transcriptional regulators; underlined : predicted transposases, integrases, recombinases

Column G: percentage of identity between the putative protein and the best CAZy homolog, according to BLASTP analysis of the putative CAZy protein against the CAZy database of characterised proteins of the same family (E-value < 10-8, query length coverage ≥50%)

Columns H & I: ORF assignment to Cluster of Orthologous Groups using RPS-BLAST analysis against the COG database (E-values ≤ 10-8).

Column J: protein ID of the best BLASTX hit of the ORF

Column K: bacterial origin of the best BLASX hit

Columns L to Q: gene occurence in the human gut microbiome determined by BLASTN comparison of the predicted ORFs with the metagenomic data sets retrieved from feacal sampling
of 163 European subjects from the MetaHit cohort (columns L and M), 139 U.S. subjetcs from the NIH-HMP cohort (columns N and O), and 110 subjects of various ages and geographical origins (columns P and Q). (E-value = 0, Identity = 90% and 100 %)
