# Supplementary material for: Functional Metagenomics Reveals Novel Pathways of Prebiotic Breakdown by Human Gut Bacteria
Source: PLoS One. 2013 Sep 16;8(9):e72766. doi: 10.1371/journal.pone.0072766 (PMC3774763; doi:10.1371/journal.pone.0072766)
Supplement: Table S2 — Glycoside hydrolase families found in the study. (DOC) [file pone.0072766.s003.doc]

**Table S2** Glycoside hydrolase families found in this study and their known activities according to the CAZy database (<http://www.cazy.org/>). In bold, activities responsible for the hydrolysis of the oligosaccharides and polysaccharides used in the screening assays.

| Glycoside hydrolase families | Known activities |
| --- | --- |
| GH2 | **β-galactosidase** (EC 3.2.1.23) ; β-mannosidase (EC 3.2.1.25); β-glucuronidase (EC 3.2.1.31); mannosylglycoprotein endo-β-mannosidase (EC 3.2.1.152); exo-β-glucosaminidase (EC 3.2.1.165) |
| GH3 | β-glucosidase (EC 3.2.1.21); **xylan 1,4-β-xylosidase** (EC 3.2.1.37); β-N-acetylhexosaminidase (EC 3.2.1.52); glucan 1,3-β-glucosidase (EC 3.2.1.58); glucan 1,4-β-glucosidase (EC 3.2.1.74); exo-1,3-1,4-glucanase (EC 3.2.1.-); α-L-arabinofuranosidase (EC 3.2.1.55). |
| GH8 | chitosanase (EC 3.2.1.132); cellulase (EC 3.2.1.4); licheninase (EC 3.2.1.73); endo-1,4-β-xylanase (EC 3.2.1.8); **reducing-end-xylose releasing exo-oligoxylanase** (EC 3.2.1.156) |
| GH10 | endo-1,4-β-xylanase (EC 3.2.1.8); endo-1,3-β-xylanase (EC 3.2.1.32) |
| GH13 | α-amylase (EC 3.2.1.1); pullulanase (EC 3.2.1.41); cyclomaltodextrin glucanotransferase (EC 2.4.1.19); cyclomaltodextrinase (EC 3.2.1.54); trehalose-6-phosphate hydrolase (EC 3.2.1.93); oligo-α-glucosidase (EC 3.2.1.10); maltogenic amylase (EC 3.2.1.133); neopullulanase (EC 3.2.1.135); α-glucosidase (EC 3.2.1.20); maltotetraose-forming α-amylase (EC 3.2.1.60); isoamylase (EC 3.2.1.68); glucodextranase (EC 3.2.1.70); maltohexaose-forming α-amylase (EC 3.2.1.98); maltotriose-forming α-amylase (EC 3.2.1.116); branching enzyme (EC 2.4.1.18); trehalose synthase (EC 5.4.99.16); 4-α-glucanotransferase (EC 2.4.1.25); maltopentaose-forming α-amylase (EC 3.2.1.-) ; amylosucrase (EC 2.4.1.4) ; sucrose phosphorylase (EC 2.4.1.7); malto-oligosyltrehalose trehalohydrolase (EC 3.2.1.141); isomaltulose synthase (EC 5.4.99.11); amino acid transporter |
| GH16 | xyloglucan:xyloglucosyltransferase (EC 2.4.1.207); keratan-sulfate endo-1,4-β-galactosidase (EC 3.2.1.103); endo-1,3-β-glucanase (EC 3.2.1.39); endo-1,3(4)-β-glucanase (EC 3.2.1.6); licheninase (EC 3.2.1.73); β-agarase (EC 3.2.1.81); κ-carrageenase (EC 3.2.1.83); xyloglucanase (EC 3.2.1.151); endo-β-1,3-galactanase (EC 3.2.1.-); β-porphyranase (EC 3.2.1.178) |
| GH32 | invertase (EC 3.2.1.26); endo-inulinase (EC 3.2.1.7); β-2,6-fructan 6-levanbiohydrolase (EC 3.2.1.64); endo-levanase (EC 3.2.1.65); **exo-inulinase (EC 3.2.1.80);** fructan β-(2,1)-fructosidase/1-exohydrolase (EC 3.2.1.153); fructan β-(2,6)-fructosidase/6-exohydrolase (EC 3.2.1.154); sucrose:sucrose 1-fructosyltransferase (EC 2.4.1.99); fructan:fructan 1-fructosyltransferase (EC 2.4.1.100); sucrose:fructan 6-fructosyltransferase (EC 2.4.1.10); fructan:fructan 6G-fructosyltransferase (EC 2.4.1.243); levan fructosyltransferase (EC 2.4.1.-) |
| GH35 | β-galactosidase (EC 3.2.1.23); exo-β-glucosaminidase (EC 3.2.1.165) |
| GH42 | **β-galactosidase** (EC 3.2.1.23) |
| GH43 | **β-xylosidase** (EC 3.2.1.37**);** β-1,3-xylosidase (EC 3.2.1.-); α-L-arabinofuranosidase (EC 3.2.1.55); arabinanase (EC 3.2.1.99); xylanase (EC 3.2.1.8); galactan 1,3-β-galactosidase (EC 3.2.1.145) |
| GH67 | α-glucuronidase (EC 3.2.1.139); xylan α-1,2-glucuronidase (EC 3.2.1.131) |
| GH77 | amylomaltase or 4-α-glucanotransferase (EC 2.4.1.25) |
| GH115 | xylan α-1,2-glucuronidase (3.2.1.131); α-(4-O-methyl)-glucuronidase (3.2.1.-) |
| GH120 | **β-xylosidase** (EC 3.2.1.37) |
